# Cellular organization and phylogeny of *Cryptonaiada* gen. n. (Cryptonaiadidae fam. n.) shed light on the evolution of intracellular vertebrate parasitism in Apicomplexa

**DOI:** 10.1128/mbio.01957-26

**Published:** 2026-10-05

**Authors:** Andrea Valigurová, Kirill V. Mikhailov, Vladimir V. Aleoshin, Natálie Glücková, Aurelie Chambouvet, Petr Papežík, Miloslav Jirků, Jan Janouškovec

**Affiliations:** 1 Department of Botany and Zoology, Faculty of Science, Masaryk University 117204, Brno, Czech Republic; 2 Department of Zoology, Faculty of Natural Sciences, Comenius University in Bratislava 724709 , Bratislava, Slovak Republic; 3 Belozersky Institute of Physico-Chemical Biology, Lomonosov Moscow State University 115375, Moscow, Russia; 4 Sorbonne Université, CNRS, UMR7144 AD2M, ECOMAP, Station Biologique de Roscoff 129716 , Roscoff, France; 5 Institute of Parasitology, Biology Centre of the Czech Academy of Sciences 446341, České Budějovice, Czech Republic; 6 Centre Algatech, Institute of Microbiology of the Czech Academy of Sciences 86863, Třeboň, Czech Republic; Universite de Geneve Faculte de Medecine, Geneva, Switzerland

**Keywords:** amphibia, tadpole, liver, macrophage, *Nematopsis temporariae*, oocyst distribution, host-parasite interactions, sporozoite ultrastructure, reclassification

## Abstract

Apicomplexan parasites have coevolved with animal hosts for hundreds of millions of years, yet only a handful of their lineages have made the transition to infecting vertebrates. Among the least understood vertebrate-infecting apicomplexans is *Nematopsis temporariae*, recently rediscovered after nearly a century of neglect. Here, we isolated this parasite from tadpole tissues and examined its host relationship, ultrastructure, and phylogenetic placement. We show that *N. temporariae*’s monozoic oocysts are persistently associated with hepatic Kupffer cells in tadpoles of several frog species and undergo maturation within them, while being absent from adult hosts. Sporozoites possess a folded multimembrane pellicle, numerous paraglycogen granules, and an apical complex with polar rings and rhoptries. Exceptionally abundant micronemes are distributed throughout the cell rather than restricted to the apical region. Although several traits superficially resemble *Nematopsis* eugregarines from marine bivalves, single- and multi-gene phylogenies demonstrate that *N. temporariae* belongs to a distinct, distantly related lineage of freshwater actinocephaloid eugregarines. This evidence warrants the establishment of the new genus *Cryptonaiada* gen. n. in the new family Cryptonaiadidae fam. n.—the first known gregarine apicomplexan to have evolved vertebrate parasitism. We propose that *Cryptonaiada* develops within macrophages upon engulfment, evading digestion, and actively modulating them through sporozoite-mediated secretion. The persistent intracellular interaction between *Cryptonaiada* and tadpole macrophages may represent an early stage in parasite coevolution with vertebrate adaptive immunity, coinciding with a window of host immunological immaturity. Our findings highlight the value of studying neglected symbionts of amphibians, a group facing a significant global population decline.

**IMPORTANCE:**

How parasites evolve to infect vertebrate hosts with adaptive immunity remains a major question in parasitology. Apicomplexans provide a unique perspective on this transition, as vertebrate parasitism has arisen in only a handful of ancient lineages. Here, we characterize *Cryptonaiada temporaria*, an early-branching freshwater apicomplexan infecting tadpoles. The evidence indicates that *Cryptonaiada* represents a new gregarine genus and family that have evolved to infect tadpoles intracellularly, via oocysts engulfed by and developing within liver macrophages. The ultrastructure of the oocyst containing a single sporozoite reveals a semi-open state combining features consistent with environmental defense and secretion into the host cell. *Cryptonaiada* is absent from metamorphosed adults, suggesting that it exploits a developmental window of reduced host immune competence. These findings reveal an unusual form of intracellular vertebrate parasitism in gregarines, providing new insight into the evolutionary transition of apicomplexans from invertebrate to vertebrate hosts.

## INTRODUCTION

Apicomplexans (Apicomplexa Levine, 1980, emended by Adl et al., 2005) are a highly diversified group of unicellular eukaryotic parasites characterized by the apical complex involved in host-cell invasion (1). Although all Apicomplexa parasitize animal hosts, only four major groups of apicomplexans occur in vertebrates: hemosporidians and piroplasms (also called Aconoidasida), eucoccidians, certain adeleorinians, and *Cryptosporidium*. These lineages have coevolved with vertebrate hosts for long evolutionary periods and evolved a wide array of adaptations to evade their adaptive immune response. Consequently, many of these vertebrate-infecting apicomplexans are of major medical and veterinary importance, causing diseases such as malaria, babesiosis, toxoplasmosis, coccidiosis, and cryptosporidiosis.

Gregarines (Gregarinasina) represent early-branching apicomplexan lineages that infect invertebrates and urochordates (2). While most gregarines have single-host life cycles, species in the family Porosporidae Labbé, 1899, have heteroxenous life cycles involving two hosts: crustaceans and mollusks (3). The porosporid life cycle involves the formation of gymnospores within gametocysts, which in molluscs develop into either naked sporozoites, as in *Porospora*, or single-sporozoite (“monozoic”) oocysts, as in *Nematopsis*. These stages typically develop within molluscan phagocytes that can migrate through connective tissues to other organs. Infections are generally associated with focal aggregation of haemocytes and usually have minimal pathological impact, although this can vary depending on the severity of the infection. The genus *Nematopsis* was established based on ovoid monozoic oocysts found in bivalves (4); its development in crustaceans was demonstrated later (5). Subsequent species from bivalves, gastropods, and chitons were often described solely on the presence of a monozoic oocyst (6, 7).

*Nematopsis temporariae* was first described over a century ago based on monozoic oocysts observed in hepatic Kupffer cells of *Rana temporaria* tadpoles (8). Although it was the only *Nematopsis* species recorded from a freshwater host, it attracted little attention for many decades. Interest in this enigmatic gregarine was renewed following its rediscovery in tadpoles of *R. temporaria*, *R. dalmatina*, and *Hyla arborea* from two geographically distant localities in Czechia (9). To date, only oocysts have been observed, precluding elucidation of its life cycle in tadpoles. No ultrastructural or host tissue distribution data are available for *N. temporariae,* and its evolutionary relationship to marine *Nematopsis sensu stricto* remained uncertain, as these taxa differ markedly in habitat, host range, and morphology.

Initial small subunit ribosomal RNA gene (SSU rRNA) phylogenies placed *N. temporariae* among “terrestrial gregarines group 1,” which corresponds to the superfamily Actinocephaloidea, with moderate confidence (9). Subsequent analyses incorporating additional actinocephaloid and marine *Nematopsis* sequences recovered the same relationship, but maximum likelihood support for it remained relatively modest (7, 10). Marine *Nematopsis* SSU rRNAs grouped closely with *Porospora gigantea*, well separated from *N. temporariae*; however, high substitution rates and poor backbone support left their broader placement among gregarines unresolved (7). Additional molecular data are therefore required to resolve the relationships of freshwater and marine *Nematopsis* species within gregarines, especially since other gregarine-like species have previously been misclassified as apicomplexans based on superficial morphology and SSU rRNA analyzes (11, 12).

Gregarines are predominantly extracellular or epicellular, and although intracellular stages occur in some lineages, sustained intracellular development in vertebrate host cells is unprecedented. The present study aims to (i) analyze the tissue distribution of *N. temporariae* in tadpoles, (ii) characterize the subcellular organization of the monozoic oocysts and their interactions with the host, and (iii) evaluate its phylogenetic position using multigene data sets. By integrating morphological and molecular evidence, we reassess the systematic placement of this enigmatic parasite and explore its implications for the evolution of vertebrate parasitism within gregarine apicomplexans.

## MATERIALS AND METHODS

### Material collection

Tadpoles of *Pelophylax esculentus* (Linnaeus, 1758), *Rana dalmatina* Fitzinger in Bonaparte, 1839, *R. temporaria* Linnaeus, 1758, *Hyla arborea* (Linnaeus, 1758), and *Bufo bufo* (Linnaeus, 1758) were collected between May and July 2004 from the locality Zaječí potok (49.23765°N, 16.60637°E), Brno, Czech Republic. Anesthetized animals were dissected and examined for the presence of parasites as described in previous studies (9, 13, 14).

### Light microscopy

Native squash preparations of tadpole tissues in amphibian Ringer’s solution (15) were examined for parasites using the Olympus AX70 light microscope equipped with Nomarski interference contrast (NIC) or Olympus BX61 microscope with phase contrast. For serial histology, anesthetized tadpoles were fixed whole in AFA (Alcohol–Formalin–Acetic Acid) fixative and processed following our modified protocol (16). Sections were stained with hematoxylin-eosin or Masson’s trichrome (fast green method) (17). Histological and toluidine blue-stained semithin sections were observed with the Olympus BX61 microscope fitted with the DP71 digital camera.

### Electron microscopy

For scanning electron microscopy (SEM), oocysts were concentrated by flotation from feces, washed three times with tap water, and stored for 2 weeks in 4% formalin. Oocysts were allowed to settle on poly-L-lysine-coated coverslips for 20 min, then fixed in 2.5% glutaraldehyde in 0.2 M cacodylate buffer for 30 min, post-fixed in 4% osmium tetroxide (1:1 with buffer) for 30 min, dehydrated, critical-point dried, and gold-coated. Samples were examined using the JEOL 6300 scanning electron microscope.

For transmission electron microscopy (TEM), parasitized pieces of liver tissue were fixed at 4°C in freshly prepared 2.5 or 5% glutaraldehyde either in 0.2 M cacodylate buffer (pH 7.4) or in 0.2 M phosphate buffer. The specimens were then washed for 1 h in the same buffer, post-fixed in 1% osmium tetroxide in the same buffer for 3 h and dehydrated in an alcohol series, before microwave-assisted embedding in Epon (Polybed 812). Sections were cut with a diamond knife and stained with uranyl acetate and lead citrate. Observations and imaging were performed using JEOL 1010 TEM.

### Transcriptome sequencing and assembly

Two parasite samples were collected for transcriptomic analysis, each from a different *B. bufo* tadpole sampled and dissected as described above: one containing approximately 10 oocysts (Nt12) and the other approximately 15 oocysts (Nt54). Samples were stored in the ATL buffer (Qiagen) at −80°C and subsequently used for RNA extraction by using the RNAqueous-Micro Kit. The total RNA was reverse-transcribed and subsequently PCR-amplified for 20 (Nt12) and 32 cycles (Nt54) by using the SMART-Seq v4 Ultra Low Input RNA Kit. Following purification, the cDNA was commercially sequenced on the Illumina NovaSeq 6000 platform (Novagene), producing 24.9M (Nt12) and 21.5M (Nt54) paired-end, 150-bp reads. The reads were adapter-trimmed and denoised in cutadapt v3.7 and assembled individually (Nt12, Nt54) and in combination (Nt12 + 54) under default settings in Trinity v2.14.0. The assembled transcripts were processed by TransDecoder v5.7.1 (18) utilizing Pfam v37.0 (19) and Swiss-Prot database (20) searches to assist with ORF prediction. The predicted peptides were further clustered by similarity using CD-HIT v4.8.1 (21) with a 90% identity threshold to reduce the redundancy of protein data sets.

### Ribosomal RNA gene phylogeny

Two ribosomal RNA gene transcripts were extracted from the Nt12 + 54 assembly by merging overlapping contigs: one comprising SSU rRNA and the other comprising 5.8S and large subunit (LSU) rRNAs. Forty-one new rRNA operon sequences of apicomplexans present as contaminants in publicly available high-throughput sequencing data of diverse invertebrates and metagenomes were retrieved (Table S1); 14 of these were assembled *de novo* from NCBI SRA reads (File S1). Phylogenetic analysis of the SSU, 5.8S, and LSU rRNA genes was performed using the pipeline described below, except that the GTR + F + I + G4 nucleotide substitution model was selected for IQ-TREE v3.0.1 and MrBayes v3.2.6 (22). A partial internal transcribed spacer 1 (ITS1) region was identified on the SSU rRNA contig and compared with published *N. temporariae* ITS1 sequences by using the same pipeline (no hypervariable site removal; TN + G4 model).

### Multigene phylogeny

The data set for phylogenomic analysis employed a curated selection of 350 orthologous groups, which were constructed earlier for studies of eukaryote phylogeny (23, 24). The initial orthogroups were identified by OrthoFinder (25) with a limited number of genomic data sets, and the selected orthogroups were then reviewed and expanded using HMMER (26) searches with the alignment profiles and reciprocal BLASTP (27) searches. Apicomplexan data sets were acquired from public repositories (Table S2) and additional assemblies and protein predictions were performed with Trinity (28) and TransDecoder (18) where necessary. For the WGS assembly of *Aggregata octopiana* (29), the predictions were carried out using the exonerate v2.2.0 program (30) by using orthologous sequences of *Toxoplasma gondii*, *Chromera velia,* and *Vitrella brassicaformis* as queries. Contaminations in transcriptomic data sets were identified with the help of DIAMOND (31) searches against the UniProt reference proteomes (20) and the TaxonKit program (32). Candidate orthologs found in the transcriptomes were examined using BLASTP searches against custom databases of potential contaminants and a database of verified apicomplexan sequences and thoroughly inspected in alignments and individual gene phylogenies. The metatranscriptomes were dominated by host tissue genes, as expected. We identified and included homologs of 40 *N. temporariae* genes in the 350-gene data set, mostly encoding ribosomal proteins, which corresponded to 5,554 sites in the final phylogenetic matrix (out of 127K). Alignments were prepared using MAFFT v7 (33) with the local pair (L-INS-i) algorithm and inspected manually using BioEdit (34). For phylogenetic inference, the reviewed alignments were processed using trimAl v1.5.0 (35) with the gap threshold of 0.1 and a custom mask designed to exclude ambiguously aligned and low-complexity regions. The trimmed alignments were then concatenated in SCaFoS (36) v1.25. Phylogenetic analysis with the concatenated 350-gene alignment was performed using the maximum likelihood implementation in IQ-TREE v3.0.1 (37). Tree reconstruction employed the LG + C60+F + G4 profile mixture model, and node support was evaluated using the ultrafast bootstrap approximation UFBoot2 (38) with 1000 replicates. The tree was visualized using MEGA v7 (39).

## RESULTS

To characterize *Nematopsis temporariae* and elucidate its interactions with the host, we sampled tadpoles of five frog species (*Pelophylax esculentus*, *Rana dalmatina, R. temporaria*, *Hyla arborea,* and *Bufo bufo*) in the Zaječí potok locality in the Czech Republic and unambiguously detected the parasite in all species. The highest infection rate was observed in tadpoles with developed hind limb buds.

This gregarine is characterized by ovoid, monozoic oocysts, which were morphologically and morphometrically consistent with the original description (8) and the previous study by some of us (9). The infected tadpoles appeared healthy, lacking any signs of disease, but some of them harbored sufficient numbers of oocysts to enable morphological and molecular analyses. Using these samples, we investigated the distribution of *N. temporariae* in host tissues, the architecture of its oocysts, and the subcellular organization of the sporozoite to identify features distinguishing it from other gregarines and to determine its evolutionary position within Apicomplexa.

### Parasite’s cellular niche and distribution within host tissues

**Fig 1:**
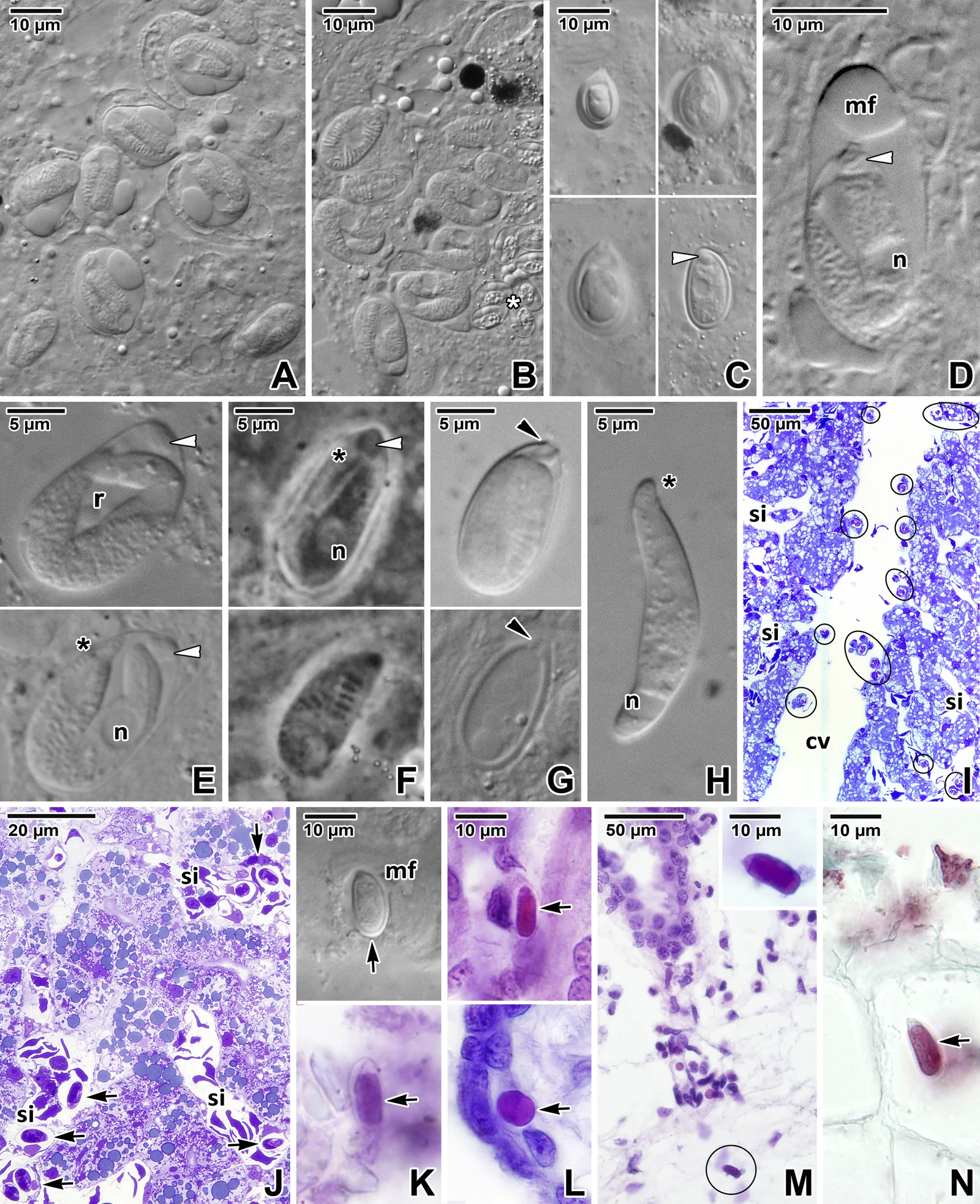
Distribution and niche of *Cryptonaiada temporaria* oocysts in tadpoles. (**A and B**) General view of liver macrophages with oocysts; co-infection with *Goussia* sp. shown in panel B. (**C**) Liver with free oocysts of different sizes. (**D**) Detail of a liver macrophage containing a single oocyst. (**E and F**) Details of oocysts within liver macrophages. (**G**) Free excysting (upper micrograph) and empty (lower micrograph) oocysts in the liver. (**H**) Free, gliding sporozoite in the liver. (**I**) General view of a liver section showing a central vein containing numerous macrophages with engulfed oocysts. (**J**) Section of liver sinusoids containing erythrocytes and macrophages with phagocytosed oocysts. (**K**) Oocyst in the gut; enclosed within a macrophage in a squash preparation (upper micrograph) and free in a histological section of the intestinal lumen (lower micrograph). (**L**) Oocyst in the gills. The oocyst in the upper micrograph is located within a macrophage. (**M**) Oocyst (encircled; detail shown in inset) within the connective tissue supporting the gills. (**N**) Oocyst in the cartilage supporting the gills. Specimen: *Bufo bufo* (**C–E** [lower micrograph], **F, I, K–N**), *Pelophylax esculentus* (**E** [upper micrograph]), *Rana dalmatina* (**A, B, G**), and *Rana temporaria* (**H, J**). LM—native squash preparation under NIC (**A–E**, **G, H**, **K** [upper micrograph]), LM—native squash preparation under phase contrast (**F**), LM—semithin sections stained with toluidine blue (**I, J**), and LM—histological sections stained with hematoxylin-eosin (**K** [lower micrograph], **L–N**). Abbreviations: black arrow, oocyst; black arrowhead, polar opening of the oocyst; black asterisk, sporozoite apex; cv, central vein; mf, macrophage; n, sporozoite nucleus; r, presumed residual body; si, liver sinusoid with blood cells; white arrowhead, sub-Stieda body-like structure; white asterisk*, Goussia* oocysts.

**Fig 2:**
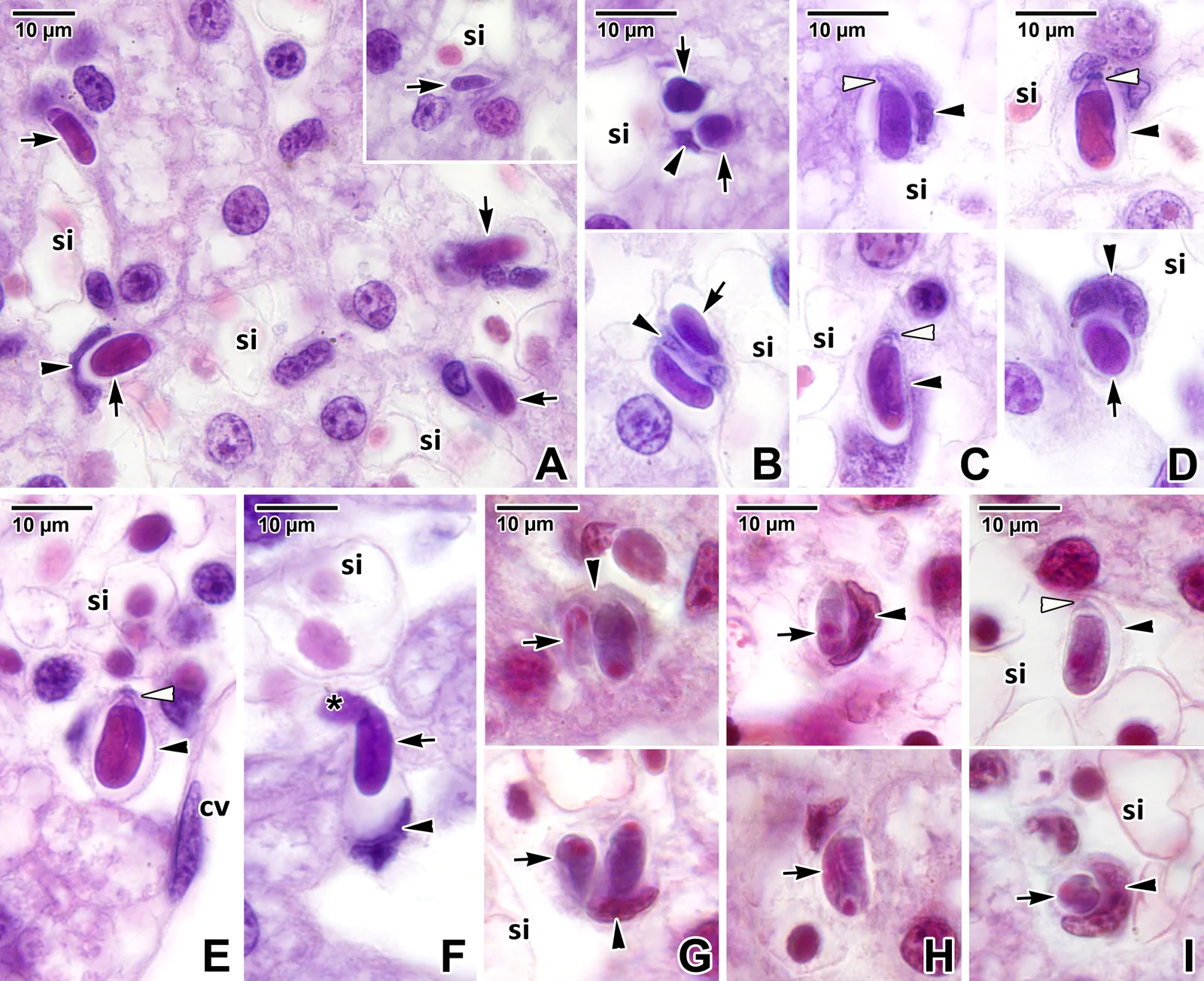
Niche and host-parasite interactions of *Cryptonaiada temporaria* in tadpole liver. (**A**) Liver section showing four differently sized oocysts. The inset shows the smallest, but already sporulated, oocyst detected in histological sections. (**B**) Pair of oocysts in cross (upper micrograph) and longitudinal (lower micrograph) sections. (**C**) Growing oocysts with their micropyles directly interacting with host macrophages. The upper micrograph shows an oocyst with a visible structure connecting its micropyle to a macrophage. (**D**) Almost fully grown oocysts in longitudinal (upper micrograph) and tangential (lower micrograph) sections. (**E**) Mature oocyst with its micropyle interacting with host macrophage. (**F**) Destroyed macrophage releasing a mature oocyst. (**G**) Pair of oocysts engulfed by a host macrophage. The upper micrograph shows two oocysts of different sizes. (**H and I**) Differently sized oocysts enclosed by intact macrophages in longitudinal (**H, I** [upper micrograph]) and tangential (**I** [lower micrograph]) sections. Specimen: *Bufo bufo* (**A–I**). LM—histological sections stained with haematoxylin-eosin (**A–F**) or Masson’s trichrome (**G–I**). Abbreviations: black arrow, oocyst; black arrowhead, macrophage; black asterisk, host cellular reaction surrounding the oocyst micropyle; *cv*, central vein; *si*, liver sinusoid with blood cells; white arrowhead, contact zone between the oocyst micropyle and the host macrophage.

To characterize the distribution of gregarine in the host, we comprehensively examined tadpole internal tissues and organs, combining native squash preparations with serial sections of whole tadpoles. Most oocysts occurred within macrophages in the liver sinusoids, at loads of one to nine per macrophage. Despite these relatively high oocyst loads, liver tissue showed no significant pathology (Fig. 1 A through G, I, and J; Fig. 2A through I); damage, if present, was confined to the host macrophages themselves. Oocysts were flattened on one side, with a funnel-like opening at the pointed end. A clear, colorless mass, resembling sub-Stieda bodies of eimeriid sporocysts and staining pale purple with toluidine blue, adhered to the narrower pole (Fig. 1D through F, I, and J). Some oocysts contained a colorless, spherical structure (Fig. 1E, upper micrograph), likely an oocyst residuum in the form of a large amylopectin granule as observed by TEM. Smaller oocysts appeared to have a more distinct wall and an underdeveloped sporozoite (Fig. 1C, upper left micrograph). Each oocyst contained a single, stout, compound sporozoite showing transverse (occasionally longitudinal) striations in squash preparations (Fig. 1A, B, and F), corresponding to micronemes arranged in parallel layers in TEM micrographs. The large, roundish nucleus was usually positioned between the middle and posterior third of the sporozoite. Rarely, free banana-shaped sporozoites were observed (Fig. 1H). These sporozoites had their nucleus located more posteriorly than the encysted forms and moved relatively rapidly by unidirectional gliding. Empty oocysts (Fig. 1G, lower micrograph) were sometimes observed freely or together with non-excysted ones within a single macrophage.

On one occasion, an oocyst was observed in a native squash preparation of the *B. bufo* tadpole intestine, and a few histological sections revealed structures resembling immature oocysts in the intestinal lumen (Fig. 1K). Serial histology of whole tadpoles also revealed the occasional presence of oocysts of various sizes in the gill epithelium, some within macrophages (Fig. 1L). Oocysts were also found in the connective tissue (Fig. 1M) and cartilage (Fig. 1N) supporting the gills.

Apart from these findings, all oocysts detected in histological sections were located in the liver sinusoids and, in most cases, within host macrophages (Fig. 2A through I). Sectioning confirmed the presence of variously sized oocysts, presumably representing different stages of oocyst maturation (Fig. 2A through D, G, and H). Paired oocysts were repeatedly observed within host macrophages (Fig. 2B and G), with some pairs exhibiting markedly different stages of maturation (Fig. 2G, upper micrograph). The micropyles of growing and mature oocysts were observed to directly interact with host macrophages (Fig. 2C through E and I), and in some cases, a tubular structure protruding from the micropyle was observed to engage with host macrophages (Fig. 2C, upper micrograph). Several oocysts were observed apparently exiting damaged host macrophages (Fig. 2F).

In the livers of three heavily parasitized *B. bufo* tadpoles with already developed hind limb buds, unidentified cells resembling various stages of aseptate, round gregarines were observed. These included single and paired cells, small clusters of cells, as well as two cells joined in a syzygy-like arrangement, and gametocyst-like forms (Fig. S1A through K). Structures strikingly resembling pairs of incompletely sporulated oocysts were also observed (Fig. S1L), further supporting the possibility that infected *B. bufo* tadpoles contained early developmental stages preceding the oocyst. Consistent with this scenario is the fact that macrophages in semithin sections from various specimens occasionally contained well-circumscribed, electron-dense masses with large amylopectin granules, which may represent sporoblasts (Fig. S1M). However, these structures were poorly preserved in ultrathin sections and therefore could not be examined at the electron microscopic level. Overall, the histological evidence highlights the consistent association of this gregarine with macrophages across multiple frog species and indicates that the parasite occurs in multiple tissues and potentially in additional developmental stages. Together with the unusual morphology of the oocyst and enclosed sporozoite, these observations suggest that the monozoic oocyst represents a specialized developmental stage rather than the complete sporogonic endpoint as known from other gregarines.

### Macrophages contain intact oocysts at different stages of maturation

**Fig 3:**
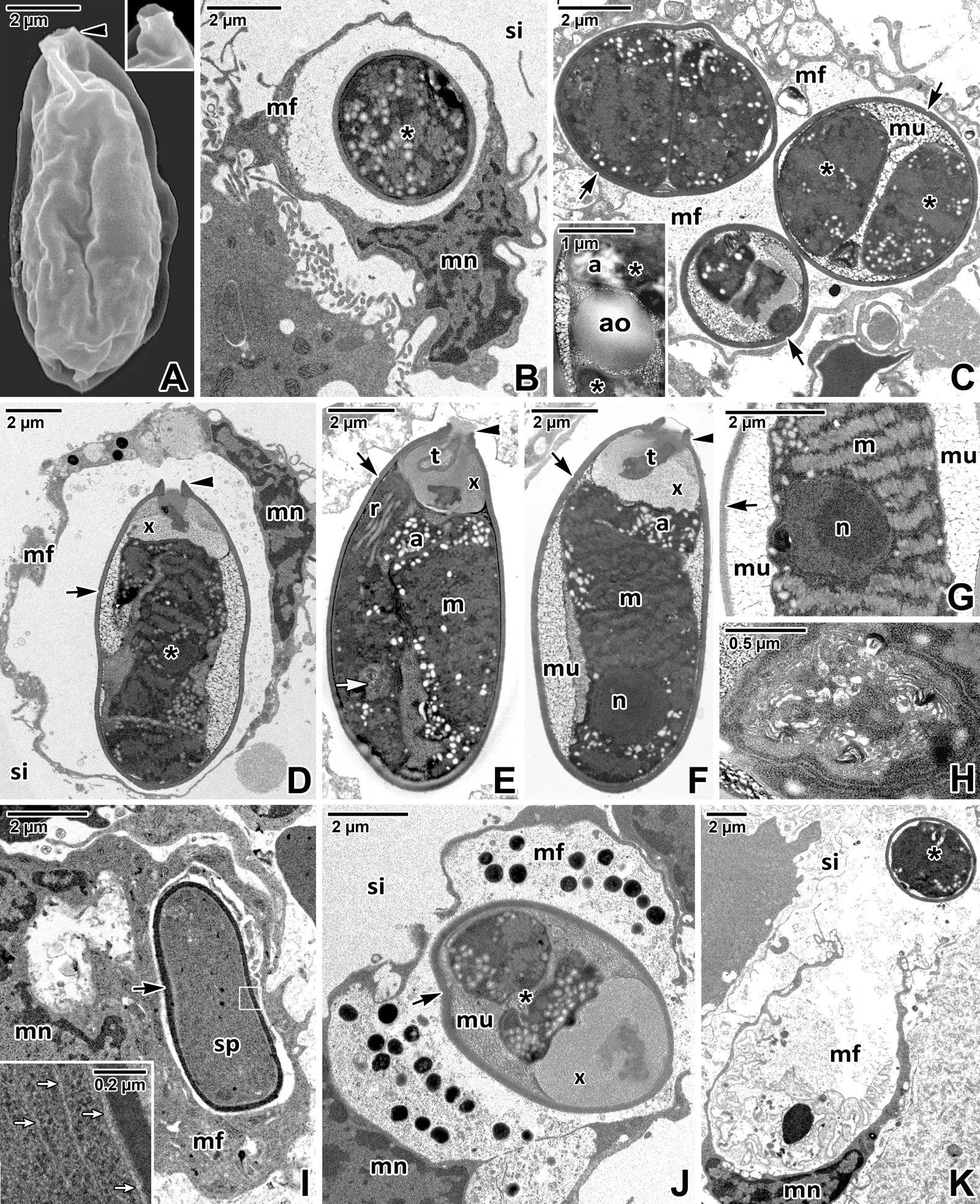
Parasite niche and architecture of *Cryptonaiada temporaria* oocysts in tadpole liver. (**A**) Oocyst; the inset shows the micropyle of another oocyst. (**B and C**) Cross-sections of a single oocyst (**B**) and multiple oocysts (**C**) within host macrophages. The inset in panel C shows a large amylopectin granule in the oocyst lumen. (**D**) Longitudinally sectioned oocyst within a macrophage. (**E and F**) Longitudinally sectioned oocysts containing single sporozoite with a prominent nucleus and abundant micronemes distributed throughout the cell. Note the multimembrane structure located in the cytoplasm of the anterior half of the sporozoite. (**G**) Detail of the sporozoite nucleus surrounded by abundant micronemes. (**H**) Detail of the unidentified multimembrane structure localized between the sporozoite apical end and nucleus. (**I**) Not fully sporulated oocyst located within a macrophage. The inset shows the detail of the immature sporozoite covered by a plasma membrane but lacking the inner membrane complex. (**J**) Damaged macrophage still containing an oocyst. (**K**) Disintegrated macrophage that has released an apparently intact oocyst. Specimen: *Bufo bufo* (**B, D, G, H, J, and K**), *Rana dalmatina* (**A**), *Rana temporaria* (**C, E, F, and I**). SEM (**A**), TEM (**B–K**). Abbreviations: a, amylopectin granules in sporozoite cytoplasm; ao, large amylopectin granule in oocyst inner space; black arrow, oocyst wall; black arrowhead, micropyle; black asterisk, sporozoite; m, micronemes; mf, macrophage; mn, nucleus of the macrophage; mu, mucous material filling the space around sporozoite; n, sporozoite nucleus; si, liver sinusoid; t, multimembrane tubular structure; white arrow, unidentified multimembrane structure; x, sub-Stieda-like body.

Light microscopy of native and histological preparations revealed oocysts as resistant, compact structures with a smooth surface. Under SEM, isolated oocysts appeared as tear-shaped sacs with a wrinkled surface and a clearly identifiable polar opening (micropyle) (Fig. 3A). Ultrathin sections confirmed the intracellular localization of the oocysts within host macrophages and revealed that they were enclosed in a parasitophorous vacuole (PV), although the PV was not always well preserved (Fig. 3B through D, I, and J). No evidence of oocyst lysis by host macrophages was found, but partially damaged (Fig. 3J) or disintegrated macrophages that had released oocysts were frequently observed (Fig. 3K). All oocysts had a thick wall lacking additional outer layers. At higher magnification, the wall appeared homogeneous, with a dense substance adhering to its inner surface (Fig. 3B through F). The large, vermiform sporozoite occupied almost the entire lumen of the oocyst and stained intensely, giving the oocyst a dense appearance. The space surrounding the sporozoite was filled with mucous material. A large amylopectin granule was also observed in a few oocysts (Fig. 3C). We also detected a small, incompletely sporulated oocyst containing a forming sporozoite covered with a still-underdeveloped pellicle (Fig. 3I), supporting histological observations of oocysts at different developmental stages within macrophages. Its wall appeared more electron-dense than that of mature oocysts. Together, this evidence suggests that host macrophages phagocytize the oocysts of this gregarine without damaging them and may host part of its life cycle.

### Giant sporozoite is protected by the oocyst wall and interacts with the host macrophage

**Fig 4:**
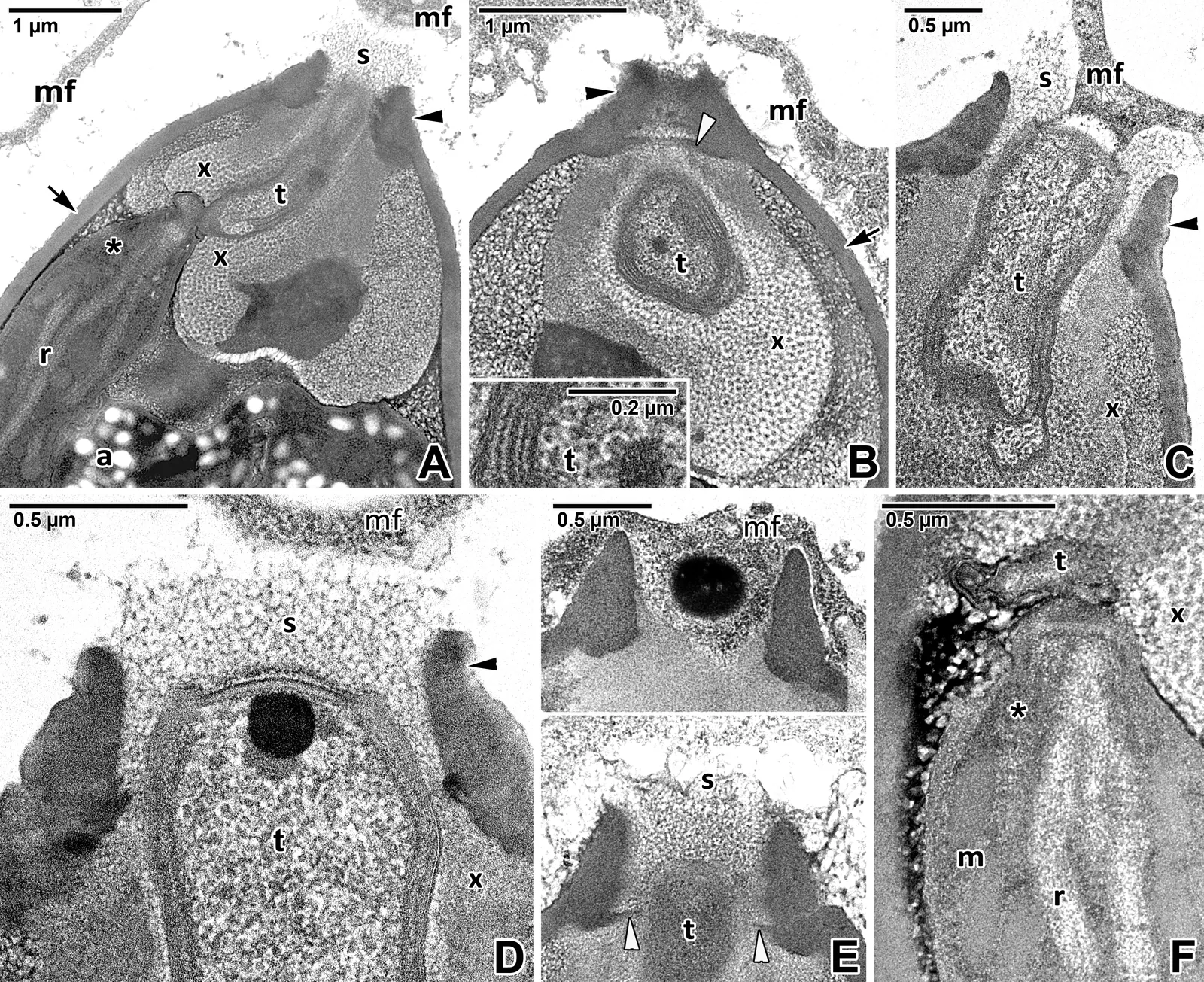
Host-parasite interactions in *Cryptonaiada temporaria* oocysts. (**A and B**) Longitudinal (**A**) and tangential (**B**) section through the pointed end of the oocyst showing the sporozoite apex attached to a multimembrane tubular structure extending into the micropyle. Note the circular barrier at the base of micropyle. The inset shows a detailed view of the membranes limiting the tubular structure. (**C–E**) Detailed views of micropyles of various oocysts directly interacting with host macrophages. Note the tubular structure passing through the circular barrier at the base of the micropyle. (**F**) Detail of the sporozoite apical end attached to the tubular structure. Specimen: *Bufo bufo* (**B–F**), *Rana temporaria* (**A**). TEM (**A–F**). Abbreviations: a, amylopectin granules; black arrow, oocyst wall; black arrowhead, micropyle; black asterisk, sporozoite’s apical end; m, micronemes; mf, macrophage; r, rhoptries; s, fibrous substance; t, multimembrane tubular structure; white arrowhead, circular barrier at the base of the micropyle; x, sub-Stieda-like body.

Of particular interest was the direct association between the pointed end of the oocyst and the host macrophage, consistently observed in both histological and semithin preparations. Ultrastructural analysis revealed that the pointed end of the mature oocyst formed a markedly protruding, funnel-shaped micropyle (Fig. 3D through F and 4A through E). A semi-dense barrier separated the oocyst lumen from the micropyle (Fig. 4B and E, lower micrograph). Different section planes showed that this barrier is a partially open, circular structure. The micropyle appeared to be open, with no evidence of a polar cap or plug. Instead, the micropyle and the subjacent area were filled with a multi-membrane, tubular structure connected to the sporozoite apex and extending through the circular barrier at the base of the micropyle (Fig. 3E and F; Fig. 4A through F; Fig. S2A through C). An electron-dense, spherical body was observed inside the micropyle, and in one ultrathin section, it was located within the tubular structure (Fig. 4D and E). A fibrous substance resembling cytoplasmic debris was observed at the interface between the micropyle and the macrophage (Fig. 4A and C through E).

**Fig 5:**
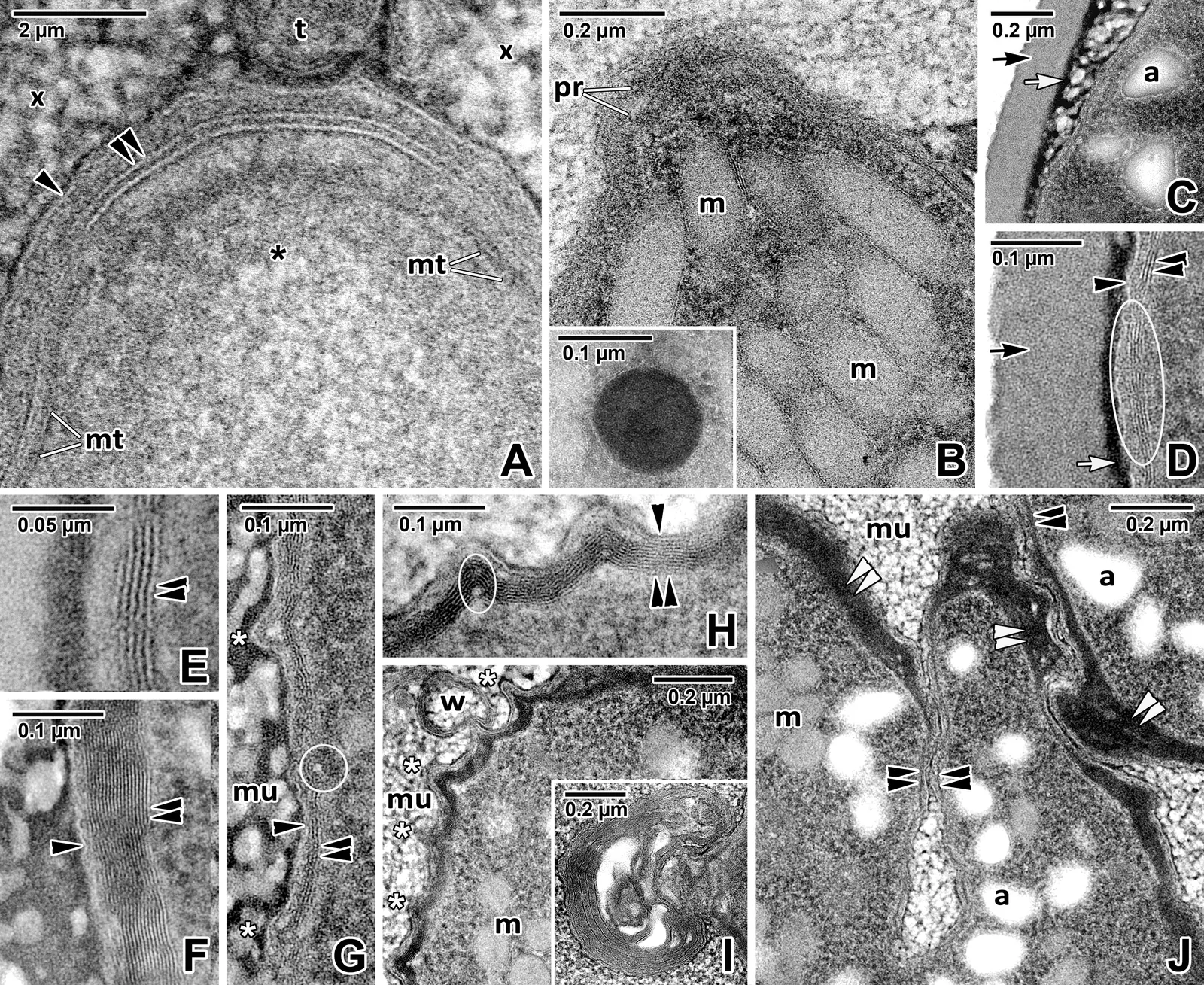
Pellicle of *Cryptonaiada temporaria* sporozoite. (**A**) The pellicle at the sporozoite apex consisting of a single plasma membrane underlain by a double-layered inner membrane complex (IMC). Note the subpellicular microtubules visible in this area. (**B**) Sporozoite apex with two polar rings. The inset shows a spherical dense structure in sporozoite cytoplasm. (**C**) Detailed view of amylopectin granules in sporozoite cytoplasm. (**D**) Sporozoite pellicle showing the double-layered IMC alternating with its multilayered regions (encircled). (**E**) Higher magnification of the IMC revealing multiple closely apposed membranes. (**F**) Pellicle with multiple membranes forming the IMC. (**G**) Folded region of sporozoite pellicle with mostly double-layered IMC. Note the translucent circular areas embedded within or below IMC. (**H**) Detail of sporozoite pellicle with distinct folding into small ridges with translucent circular areas. (**I**) Membrane whorls associated with the folded pellicle. (**J**) General view of the sporozoite pellicle with localized thickenings due to multiple IMC layers. *Bufo bufo* (**B, D–G**), *Rana temporaria* (**A, C, H–J**). TEM (**A–J**). Abbreviations: a, amylopectin granule; black arrow, oocyst wall; black arrowhead, sporozoite plasma membrane; black asterisk, sporozoite cytoplasm; double black arrowhead, IMC; double white arrowhead, areas of thickened IMC; m, micronemes; mu, mucous material filling the space around sporozoite; r, rhoptry; t, multimembrane tubular structure; w, membrane whorl; white arrow, dense layer covering the inner side of the oocyst wall; white asterisk, pellicular ridge, x, sub-Stieda-like body.

The inner space of the oocyst’s pointed end was filled with granular material (Fig. 3D through F and J and 4A through D), corresponding to the colorless mass resembling the sub-Stieda body observed under light microscopy. The sporozoite apex extended into the space of this structure (Fig. 4A; Fig. S1A through C). The apical end of the sporozoite contained two polar rings and was packed with several long rhoptries (Fig. 3E; Fig. 4A and F; Fig. 5B). No conoid was observed. Longitudinally oriented subpellicular microtubules could be unambiguously identified only in the region of the apical end (Fig. 5A). The sporozoite cytoplasm was packed with an unprecedented number of micronemes and amylopectin granules, and a few dense spherical structures bounded by a single membrane (Fig. 3E through G, J;, Fig. 4A; Fig. 5B, C, and J). A nucleus with a single prominent nucleolus, usually located in the region of sporozoite folding, was surrounded by the endoplasmic reticulum. An unspecified multi-membranous structure was repeatedly observed in the anterior middle of the sporozoite, located in between its apical end and nucleus (Fig. 3E and H). The sporozoite pellicle exhibited variable thickness of the inner membrane complex (IMC). In the thinner regions, a typical three-layered apicomplexan pellicle was present, consisting of the plasma membrane and a double-layered IMC (i.e., flattened cortical alveoli, comprising external and internal cortical cytomembranes) (Fig. 5A and C). In thickened regions, the cortical cytomembranes were further multiplied (Fig. 5D through J). The plasma membrane was uniformly single-layered. Tangential and superficial sections revealed the sporozoite cell coat and unusual folding of its pellicle, forming small ridges (Fig. 5G through J). Translucent circular areas with dense peripheries (7–9 nm in diameter) were located beneath the two-layered IMC. In the folded pellicle regions with multiplied cortical cytomembranes, these circular structures appeared embedded within the IMC (Fig. 5H). Membrane whorls were frequently associated with the sporozoite pellicle (Fig. 5I). Together, the ultrastructural evidence reveals that liver macrophages host gregarine oocysts containing maturing or mature sporozoites, which possess a secretory gateway with polar rings, rhoptries and an overabundance of micronemes, indicating active secretion potential for interaction with the host cell.

### Phylogenetic evidence supports a new family and independent origin of vertebrate parasitism in gregarines

We next aimed to remove any remaining ambiguity about *N. temporariae*’s phylogenetic position to understand the evolutionary context in which it developed an association with vertebrates and reassess its classification. We used a low-input RNA-Seq protocol to sequence two high-throughput metatranscriptomes from *B. bufo* tadpole liver samples containing approximately 10 and 15 *N. temporariae* oocysts. The two samples contained genetically identical rRNAs of *N. temporariae*, so we generated a combined assembly of all reads and examined its relationships by rRNA and multigene phylogenies (see Materials and Methods).

**Fig 6:**
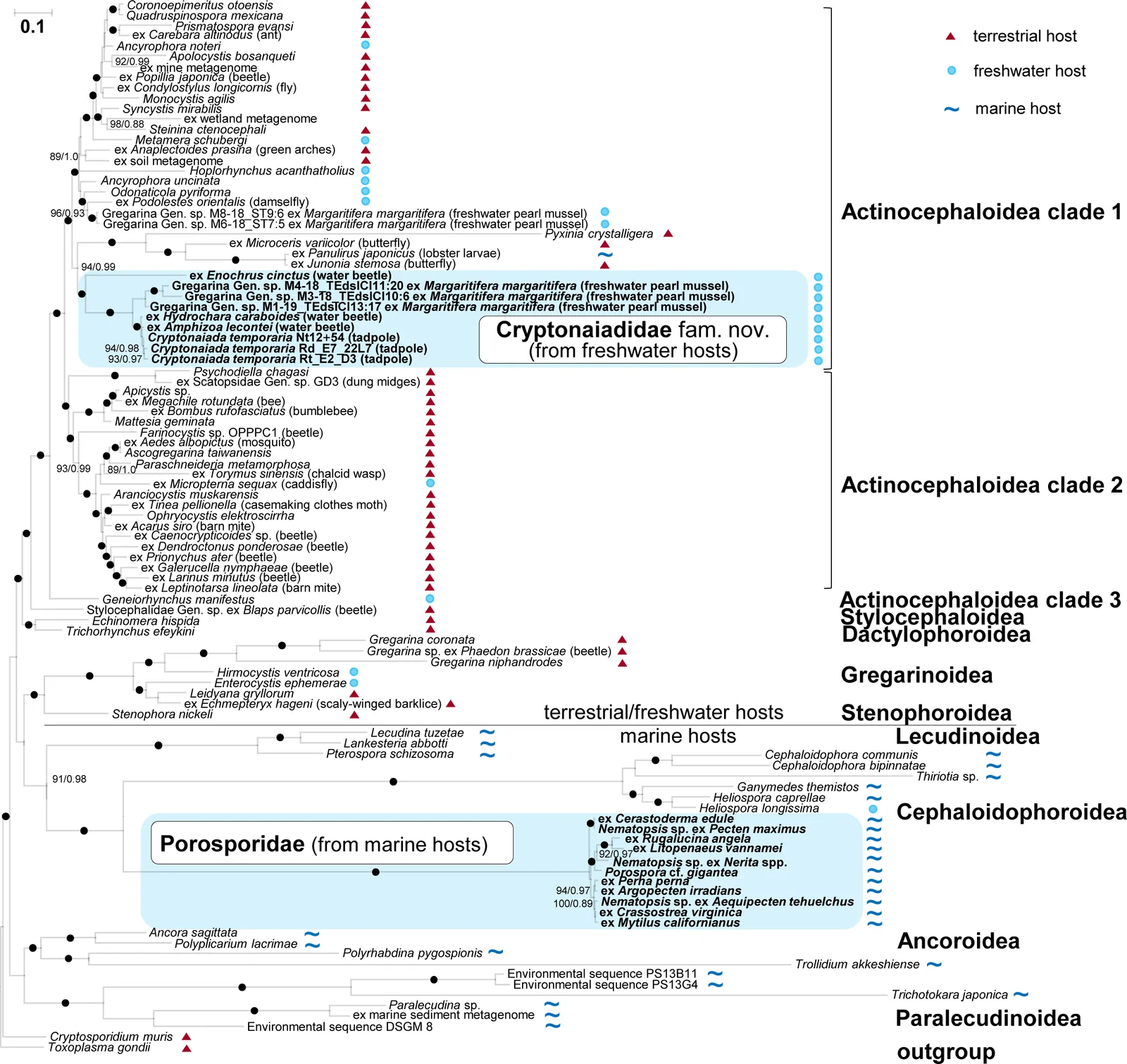
Phylogeny of the ribosomal RNA operon supports the erection of a new apicomplexan family. Branch supports correspond to maximum likelihood bootstraps (IQ-TREE with the GTR + F + I + G4 model)/Bayesian posterior probabilities (MrBayes, the same substitution model). Values greater than 95/0.95 are shown by black dots, and values below 85/0.85 have been omitted.

The rRNA data set was built from representative apicomplexan rRNA operon sequences, combining SSU rRNA, as the standard apicomplexan classification marker, with 5.8S and LSU rRNAs (Table S1; see Materials and Methods). A near-complete *N. temporariae* rRNA operon, split across two contigs, was obtained from the combined assembly. To improve sequence presence, we extracted 41 new fragments of rRNA operon sequences of gregarines, primarily actinocephaloid, as contaminants in publicly available high-throughput sequencing data of diverse invertebrates and metagenomes (wgs, TSA, and SRA NCBI databases) and metagenomes, including 14 new assemblies from raw reads (File S1). Consequently, the set of complete or partial gregarine LSU rRNA genes in the alignment expanded by 41 species, including close relatives of marine *Nematopsis* species. The final rRNA operon data set had markedly better sequence presence, number of informative sites, and tree resolution than published SSU rRNA trees (Fig. 6). *Nematopsis temporariae* branched with strong support among actinocephaloid gregarines, while marine *Nematopsis* grouped, also with strong support, among cephaloidophoroids. Several environmental sequences closely related to *N. temporariae* were derived from freshwater rather than terrestrial hosts, unlike the majority of formally described actinocephaloids (Fig. 6). These relatives comprised the recently described parasite of pearl mussels (10) and three sequences from water beetles, two of which were near-identical to *N. temporariae*. A partial sequence of the *N. temporariae* internal transcribed spacer 1 (ITS1) clustered with one of the two ribotypes previously identified by Chambouvet et al. (9), which we define here as ribotype A (Fig. S3). No other ribotype was identified in our transcriptome, suggesting that ribotypes A and B represent different genotypes rather than divergent rRNA gene copies within a single genome. Both ribotypes were distributed across different frog species and a new ribotype C was identified in an environmental sequence from Quebec (accession KIHL01076253.1; Fig. S3).

**Fig 7:**
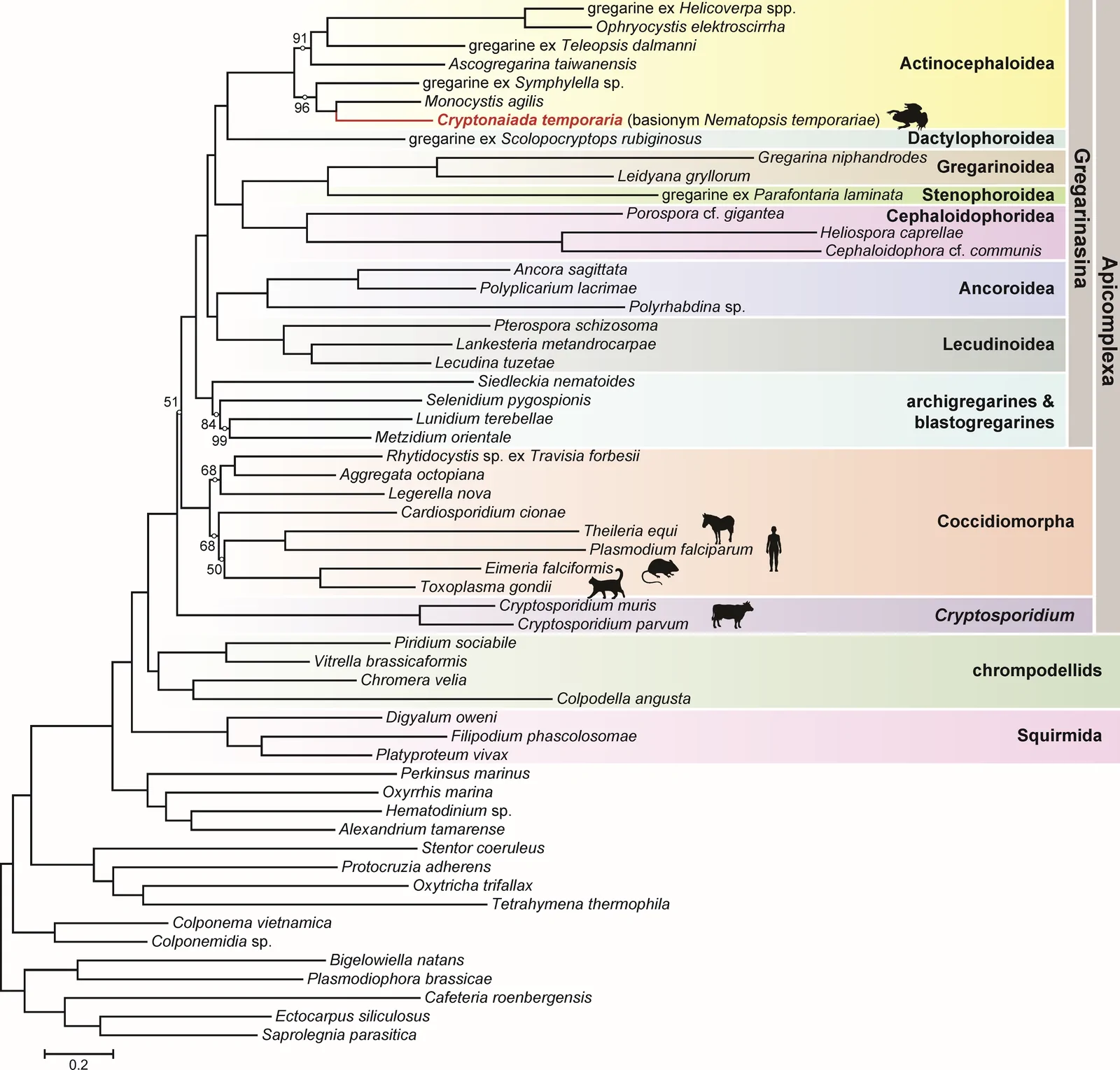
Phylogeny of 350 genes supports an independent origin of vertebrate parasitism in gregarines. The maximum likelihood phylogeny was inferred in IQ-TREE under the LG + C60 + F + G4 profile mixture model. Branch supports correspond to ultrafast bootstraps with 1,000 replicates; only values below 100% are shown, and the corresponding branches are marked with white dots. Pictograms of animals were obtained from PhyloPic (https://www.phylopic.org/).

The multigene data set was prepared by merging *N. temporariae* protein sequences into a new phylogenetic matrix of 350 orthogroups containing 33 representative apicomplexans and 22 related eukaryotes from the SAR supergroup (Fig. 7; Table S2; see Materials and Methods). The maximum likelihood phylogeny, inferred under the LG + C60+F + G4 model, was consistent with published studies and recovered monophyletic Apicomplexa, Gregarinasina, and major gregarine superfamilies with full support at nearly all internal branches (11, 29). The phylogenetic analysis placed *N. temporariae* deep among actinocephaloid gregarines with maximal support. In agreement with the rRNA phylogeny, *N. temporariae* was closest to *Monocystis agilis*, isolated from an earthworm, whose trophozoites and gamonts likewise lack a septum, as do the gregarine-like cells detected in tadpole liver. It was further related to an unidentified gregarine from the myriapod *Symphylella*. In contrast, *Porospora*, the close relative of marine *Nematopsis* species, grouped with full support among cephaloidophoroid gregarines, as in the rRNA tree (Fig. 6 and 7). No transcripts of apicoplast proteins were identified.

These findings establish *N. temporariae* as the only known gregarine able to infect vertebrate hosts. Support for *N. temporariae* as a member of a new lineage of freshwater actinocephaloids, far removed from marine *Nematopsis* species, justifies the erection of a new genus *Cryptonaiada* gen. n. and family Cryptonaiadidae fam. n. within gregarine apicomplexans.

### Taxonomic summary

We reassess the taxonomic position of the species *Nematopsis temporariae* Nöller, 1920, and transfer it to the newly established genus *Cryptonaiada*, placing it within the new family Cryptonaiadidae.

Phylum Apicomplexa Levine 1980, emend. Adl et al. 2005

Class Conoidasida Levine 1988

Subclass Gregarinasina Dufour 1828

Order Eugregarinorida Léger 1900

#### Cryptonaiadidae fam. n.

ZooBank registration number of present work: urn:lsid:zoobank.org:pub:E983659F-2A58-46EC-B589-784AA87FB889.

ZooBank registration number of new family: urn:lsid:zoobank.org:act:57AC8C28-8FED-406D-B115-2849FAEA59BB.

Diagnosis: Eugregarine apicomplexans occurring in freshwater environments, which form a well-supported monophyletic clade in ribosomal RNA operon phylogeny defined as the least inclusive clade containing *Cryptonaiada temporaria* (accession OZ557594) and the sequence of an unidentified gregarine ex *Enochrus cinctus* (accession JAUIMW010012868.1). Members of the family are distinct from other families of Eugregarinorida by their phylogenetic placement.

Type genus: *Cryptonaiada* gen. n.

Etymology: Formed by adding the family-group suffix -idae to the stem of the type genus name (Articles 29.1 and 29.2 of the ICZN, 1999).

Remarks: The family Cryptonaiadidae comprises one formally established genus, *Cryptonaiada* gen. n. Additional probable representatives include an undescribed gregarine whose cysts with vermiform sporozoites were obtained from freshwater pearl mussels (*Margaritifera margaritifera*) collected in Sweden (10). Three additional sequences from aquatic beetles (deposited in File S1 of this study) group within this clade; however, the morphology and life cycle stages of the corresponding organisms are currently not known. Phylogenetic analyses of ribosomal sequences place this lineage as a distinct clade within Actinocephaloidea, separate from previously described families. No distinctive morphological synapomorphies are currently known.

#### *Cryptonaiada* gen. n.

ZooBank registration number of new genus: urn:lsid:zoobank.org:act:F15695D1-E1E1-4667-A163-B209782D8904.

Diagnosis: Oocysts occur in macrophages within the hepatic sinusoids of frog tadpoles. Oocysts are ovoid, containing a single sporozoite (monozoic). No operculum is present. The sporozoite fills almost the entire lumen of the oocyst and is folded twice, with its anterior end directed toward the micropyle. The sporozoite often appears transversely striated due to abundant micronemes arranged in parallel layers. The cytoplasm contains numerous amylopectin granules and a nucleus with a single prominent nucleolus, generally positioned within the folded region of the sporozoite or between its middle and posterior thirds.

Type species: *Cryptonaiada temporaria* (Nöller, 1920) comb. n.; basionym: *Nematopsis temporariae* Nöller, 1920 (previous combination: *Haemogregarina temporariae* (Nöller, 1920) Levine and Nye, 1977) (8, 40).

Etymology: A compound noun formed from the stem of the Ancient Greek adjective *krŭptós* (*κρῠπτός*; hidden, concealed), the thematic vowel -*o*-, and the Latinized noun *Naiada*, derived from the Greek *Nāïas* (*Ναϊάς*; water nymph, river nymph, referring to young, beautiful women inhabiting freshwater in Greek mythology). The new genus-group name alludes to the connection of the type species with the freshwater habitat. *Cryptonaiada* is treated as feminine (Article 30.2.2 of the ICZN, 1999).

Remarks: Levine and Nye (40) reassigned the basionym (*Nematopsis temporariae*) to the genus *Haemogregarina* without providing justification for this taxonomic change. The phylogenetic analyses in the present study do not support this placement, and we find no genetic or morphological evidence linking *C. temporaria* to *Haemogregarina*.

## DISCUSSION

Ancestral apicomplexans likely parasitized and diversified among marine invertebrates before colonizing freshwater and terrestrial invertebrates, and eventually vertebrates (41). The general correlation between parasite phylogeny and the taxonomy of their hosts suggests that apicomplexans ancestrally had invertebrate definitive hosts and their heteroxenous life cycles evolved independently in response to changes in host feeding behavior (42). Four well-characterized heteroxenous lineages are known to infect vertebrate hosts: hemosporidians and piroplasms, eucoccidians, some adeleorinians, and *Cryptosporidium*. In addition, the recently described but yet uncharacterized intracellular parasites of fish (“ichthyocolids”; 43) have either a shared or independent origin of vertebrate parasitism with respect to eucoccidians. This study provides a taxonomic revision and the first ultrastructural analysis of *Cryptonaiada temporaria* (basionym *Nematopsis temporariae*) and supports it as the only known gregarine infecting a vertebrate host.

### *Cryptonaiada* belongs to a new family of freshwater actinocephaloid gregarines

*Cryptonaiada temporaria* is clearly phylogenetically distant from the family Porosporidae Labbé, 1899, where this species was previously classified (3, 7). Members of this family have a marine heteroxenous life cycle, with vegetative development in the digestive tract of decapod crustaceans (extracellular septate trophozoites, caudofrontal syzygy, encystation) and sporogony in molluscan connective tissue (3, 6). The heteroxenous life cycle of *Nematopsis* Schneider, 1892 remains unclear, mainly due to differing interpretations of its sporogony. In *N. ostrearum*, it occurs in decapods, producing gymnospores that infect mollusks and form resistant oocysts within host leucocytes; in *N. legeri*, gametogenesis in decapods produces heliospores that infect mollusk gills, where they are captured by phagocytic leucocytes and develop into infective naked sporozoites (3, 6). During our study of *C. temporaria*, we observed oocysts at various stages of maturation but did not find any structures resembling heliospores or gymnospores within tadpole macrophages.

The position of *Cryptonaiada* among apicomplexans was partly resolved in published SSU rRNA phylogenies, which generally showed the parasite branching among “terrestrial gregarines clade 1” with medium strong support (7, 9, 10). Multigene and rRNA operon phylogenies in this study identify *C. temporaria* specifically as an actinocephaloid gregarine with complete support. We also show that it clusters with several sequences of symbionts of freshwater invertebrates (Fig. 6 and 7). Only one of these symbionts has been morphologically characterized: an undescribed parasite with cysts and vermiform zoites from the gills and other organs of diseased freshwater pearl mussels (10). The number of zoites per cyst in this gregarine ranged from one in the digestive gland, through one to two in the hemolymph sinuses of the mantle to many in the endothelium of the gill vessels. *In situ* hybridization documented histozoic cysts and zygote or sporoblast-like structures in digestive gland tissues (10). We observed similar structures in the intestinal wall, gills, liver, and other tissues of the tadpoles, but did not attribute them to *C. temporaria* in the absence of specific tests, because their morphology did not resemble any known stages of gregarines. Nevertheless, our understanding of the life cycle of *C. temporaria* and the mussel parasite is incomplete, and we therefore cannot easily compare their developmental stages, especially since it is possible that the life cycle of *Cryptonaiada* also includes an invertebrate host. Two SSU rRNA sequences near-identical to *C. temporaria* were retrieved from high-throughput sequence data of water beetles (Fig. 6), suggesting they likely ingested oocysts in the water column.

Grown tadpoles with hind limb buds showed the highest abundance of *C. temporaria* oocysts, together with the presence of round gregarine-like cells. If these cells represent trophic or sexual stages of the parasite, as our observations suggest, they could facilitate parasite spread through the production of dissemination and infective stages (gametocysts, oocysts). At this stage of development, tadpoles increasingly swim to the water surface and gulp air, behavior that may elevate their exposure to predators. In aquatic habitats, tadpoles are commonly preyed upon by predatory insects such as dragonfly larvae (Odonata), predaceous diving beetles (Dytiscidae), and water bugs (Nepomorpha) (44–46). Several other gregarines from freshwater invertebrates are known and have recently been sequenced (47), although none of them is specifically related to Cryptonaiadidae (Fig. 6). Predation by freshwater invertebrates could similarly facilitate transmission of oocysts or other infective stages of *C. temporaria* between tadpoles and invertebrate hosts as a part of a heteroxenous life cycle. Alternatively, predatory insects may merely serve as a means of spreading the infectious stages of the parasite over long distances.

### Similarities between *Cryptonaiada* and *Nematopsis* are due to convergent evolution

**Fig 8:**
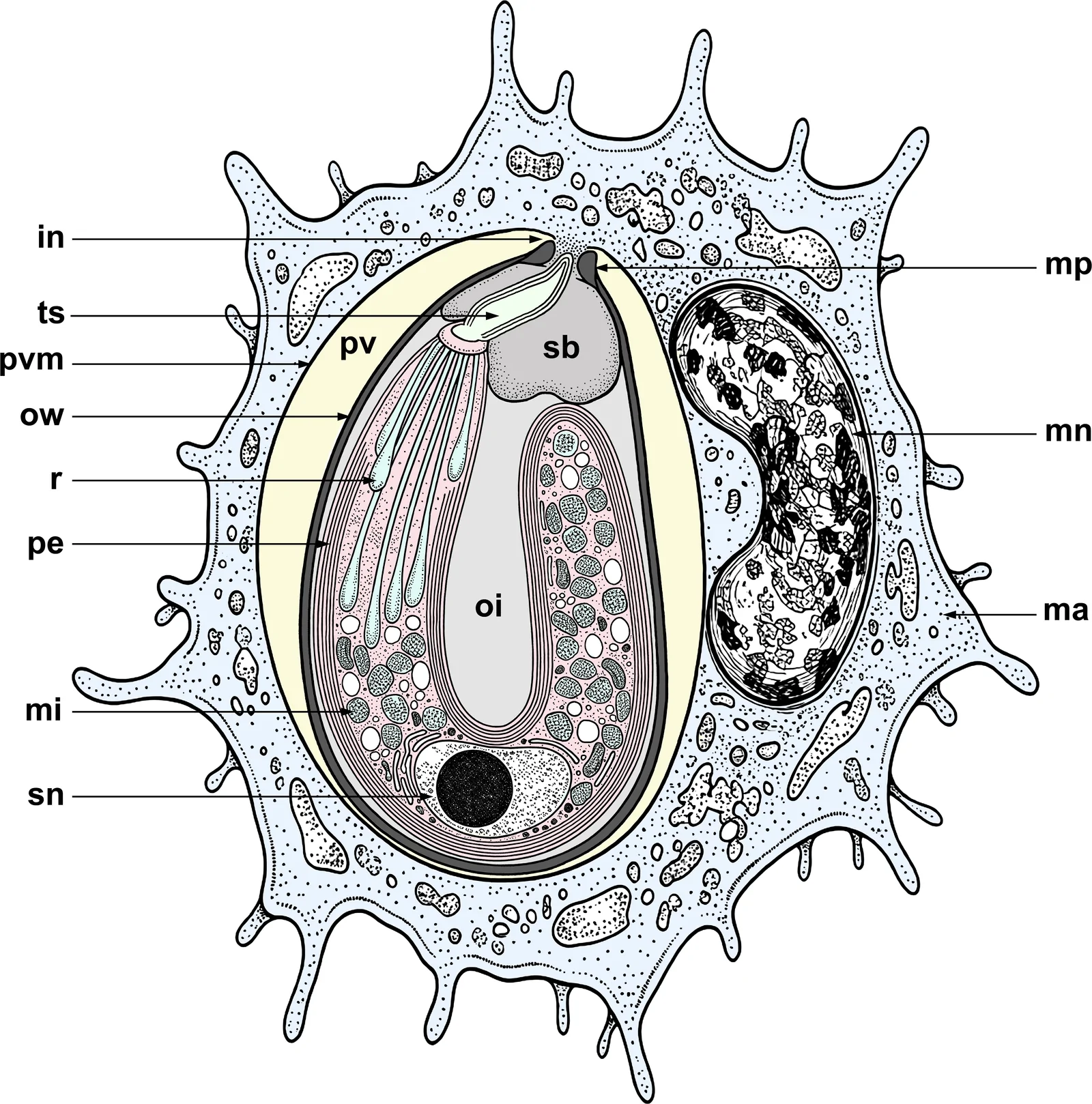
Schematic overview of host–parasite interactions and subcellular organization in *Cryptonaiada temporaria*. Abbreviations: a*,* amylopectin granule; in, site of intimate interaction between the host macrophage cytoplasm and parasite, m, macrophage; mi, microneme; mn, macrophage nucleus; mp, micropyle; oi, inner space of the oocyst; ow, oocyst wall; pe, pellicle with a multilayered inner membrane complex; pv, parasitophorous vacuole; pvm, membrane of the parasitophorous vacuole; r, rhoptry; sb, sub-Stieda-like body; sn, sporozoite nucleus; ts, multimembrane tubular structure.

Gregarine oocysts conventionally enclose several sporozoites and function primarily as resistant transmission stages (6). In contrast, *C. temporaria* and *Nematopsis* have monozoic oocysts with a giant folded zoite that are engulfed and disseminated through the host tissues by phagocytes, where they mature within a PV to produce a single, compound sporozoite (3, 6, 48). In both genera, one or multiple oocysts may be found within a single macrophage (49, 50). Observations of oocyst excystation (51) show that the *Nematopsis* sporozoite has a morphology and pellicle ultrastructure (presence of small ridges) similar to sporozoites of *C. temporaria*. Sporozoite cytoplasm in both genera is densely packed with amylopectin granules and micronemes. The multi-membrane tubular structure connecting the apex of the *C. temporaria* sporozoite with micropyle (Fig. 8) may be functionally similar to the dense structure that connects the sporozoite and micropyle in *Nematopsis* (49, 52, 53). Finally, the fibrous connection between the micropyle of the *C. temporaria* oocyst and the PV may be analogous to the dense cord connecting the oocyst cap and the PV in *Nematopsis* (7, 49, 52). This specialized micropyle-associated secretory architecture appears to maintain structural communication with the host macrophage. It is likely that the monozoic oocyst in *Cryptonaiada* and *Nematopsis* represents a specialized developmental stage, rather than the canonical terminal sporogonic product of other gregarines. In *Cryptonaiada*, this stage may undergo further development within tadpole macrophages, giving rise to other stages (Fig. S1), potentially completing the parasite’s life cycle in the vertebrate host.

These morphological and developmental similarities make it unsurprising that *Cryptonaiada* and *Nematopsis* were once placed together in the same genus, but molecular evidence now questions whether these traits reflect common ancestry or independent origins. The most recent common ancestor of the two parasites gave rise to many other eugregarines, which lack these traits—many independent losses across multiple superfamilies would be required under a scenario of vertical descent (Fig. 6 and 7) (7, 9). We therefore posit that shared similarities emerged through convergent evolution. The reasons for such convergence may relate to analogous infection routes in tadpole and bivalve hosts, in which infective stages are ingested by host phagocytes and subsequently develop within them. Indeed, as in *Nematopsis* (6), a few smaller oocysts of *C. temporaria* were observed in the gills and associated connective tissue, which may suggest a similar route of infection. In their natural habitats, bivalves filter-feed through their gills, while often omnivorous tadpoles graze on biofilms and suspended particles, providing analogous routes of exposure to waterborne parasites. Consequently, both bivalves and tadpoles host similar groups of parasites, including perkinsids, gregarines, and trichodinid ciliates, and both can serve as intermediate hosts for trematodes (54, 55).

The most obvious difference from *Cryptonaiada* is the pathogenicity of *Nematopsis* infection*,* which is characterized by tissue lysis and even mortality of the bivalve hosts, especially at high infection rates (48). The pathology is possibly linked to the complex network of microfibrils (apparently absent in *Cryptonaiada*)*,* which extends from the oocyst wall to the PV periphery, and appears to facilitate destruction of the host cell (49, 50, 56). Altogether, however, the degree of similarity between *Cryptonaiada* and *Nematopsis* makes it one of the most striking examples of convergent evolution currently recognized among apicomplexans.

### On the way to a vertebrate host

Most *C. temporaria* oocysts were found inside Kupffer cells, the dominant macrophages of the tadpole liver. No macrophage-induced damage to oocysts or the embedded sporozoites was observed; instead, oocysts varied in size, staining, and ultrastructural properties, consistent with their development within the host cell. Unusually, the micropyles of all oocysts were partially open and in direct contact with host macrophages. Apicomplexan oocysts normally carry a cap (operculum), so this exposed configuration was unexpected. At the micropyle, we observed signs of damage to the PV membrane and direct contact with the macrophage cytoplasm (Fig. 4C through E). In addition, we found oocysts released from disintegrated macrophages and several free, rapidly moving sporozoites in native squash preparations (Fig. 1H), although we cannot exclude that some of these observations stemmed from mechanical damage to macrophages. Collectively, these observations suggest that *C. temporaria* sporozoites develop within and actively interact with host macrophages through the semi-open micropyle (Fig. 8).

**Fig 9:**
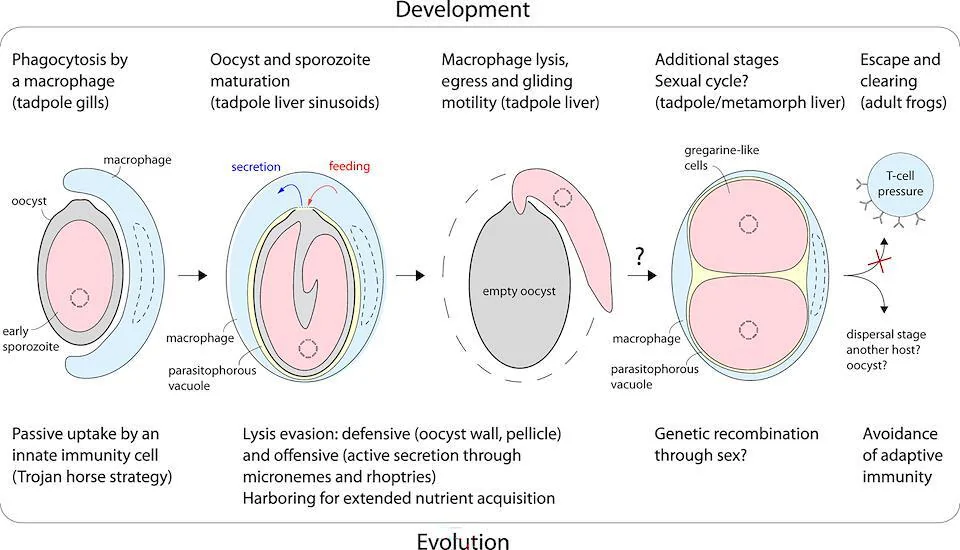
Schematic model combining the proposed life cycle and evolutionary innovations of *Cryptonaiada temporaria*. The upper row depicts the parasite’s hypothesized developmental trajectory in tadpoles: phagocytosis by a macrophage, oocyst and sporozoite maturation in the liver sinusoids, macrophage lysis and egress, possible additional stages including a sexual cycle, and eventual escape and clearance in adult frogs under T-cell pressure. The lower row presents the corresponding evolutionary interpretation of each stage, including passive uptake by an innate immune cell ("Trojan horse" strategy), lysis evasion through defensive (oocyst wall, pellicle) and offensive (micronemes, rhoptries) mechanisms, extended nutrient acquisition through intracellular harboring, possible genetic recombination, and dispersal as a mechanism for avoiding the adaptive immune barrier.

Several ultrastructural features of the sporozoite are consistent with this interpretation. The cytoplasm of *C. temporaria* sporozoites is densely packed with micronemes (Fig. 8). Their unusually high abundance suggests elevated protein secretion, which in apicomplexan sporozoites is typically linked to gliding motility, attachment, interaction with cell surface receptors, and host cell entry (2). The sporozoite also contains abundant amylopectin granules, which function as long-term energy reserves in apicomplexans. These reserves are particularly important in apicomplexan sporozoite and bradyzoite stages, in which they support survival during environmental stress or prolonged infection (57). Consistent with the metabolic streamlining characteristic of eugregarines (11, 58–60), neither morphologically identifiable apicoplast nor transcripts encoding apicoplast proteins were detected. An unknown multimembrane structure was present between the apical end and the nucleus of the *C. temporaria* sporozoite, but lacked characteristic apicoplast features (Fig. 3E and H). Finally, the unique, multilayered pellicle of the sporozoite, often folded into small ridges, may represent a specific adaptation to life within phagocytic cells—a niche atypical for gregarines (marine *Nematopsis* being a notable exception). This elaborate pellicle may maintain sporozoite structural integrity and facilitate rapid movement through host tissues. Together with the apparent ability to secrete proteins while remaining protected within a semi-open oocyst, these features may represent adaptations that enable *C. temporaria* to persist within vertebrate macrophages (Fig. 8 and 9). Combined with its low infection pathogenicity, these traits may have enabled *C. temporaria* to exploit a novel vertebrate niche while avoiding elimination by the host immune system.

Similar exploitation of macrophages has been described in other host–pathogen systems. For example, *Leishmania* spp. establish intracellular infections by subverting macrophage defense machinery, thereby creating a protected niche for parasite survival and replication (61). Likewise, ranaviruses infect macrophage-lineage cells, which serve as reservoirs for viral persistence and vehicles for dissemination within the host. In *Xenopus laevis*, macrophages have been shown to harbor dormant viral genomes that can be reactivated during inflammation, highlighting their dual role as both immune effector cells and targets of pathogen exploitation (62). This exploitation of professional phagocytes as protected intracellular niches and vehicles for pathogen dissemination has been described as a “Trojan horse” strategy. The apicomplexan *Toxoplasma gondii*, for example, exploits migratory immune cells to promote parasite dissemination through host tissues (63). As in *Leishmania* and, probably, ranaviruses, macrophage entry in *C. temporaria* likely occurs via host-mediated phagocytosis of the oocyst rather than active, parasite-driven invasion. Although the molecular mechanisms underlying infection are undoubtedly different between these organisms, they may share a common principle in which pathogens co-opt professional phagocytes to evade immune clearance and facilitate dissemination. Because macrophage-mediated digestion of cellular material is also a prime source of nutrients, harboring within them could provide parasites with a key advantage for dissemination. *Cryptonaiada temporaria* may therefore represent an analogous case—combining passive uptake by macrophages with evasion of intracellular degradation and prolonged intracellular persistence—which may represent an important early step in the evolution of vertebrate parasitism (Fig. 9).

Another important aspect of parasite development and evolution is the location of the sexual cycle. We observed spherical gregarine-like cells and syzygy-like associations inside liver macrophages of three different *B. bufo* tadpoles in addition to oocysts and sporozoites. These cells may represent additional developmental stages of *C. temporaria*, such as trophozoites, solitary and paired gamonts, and gametocysts (Fig. S1A through K). Although these cells preserved poorly in ultrathin sections, making their precise identification difficult, to our knowledge no such cells of host or parasite origin have previously been reported in tadpole liver (8, 13, 14, 64–66). Only fully sporulated oocysts of the distantly related coccidian apicomplexan *Goussia* were found in macrophages in liver sinusoids (Fig. 1B), while other developmental stages of *Goussia* are restricted to the tadpole intestine (66). Indeed, the spherical gregarine-like cells differ morphologically from the endogenous stages of any known coccidian and were consistently located alongside *C. temporaria* oocysts, some of which displayed characteristics of sporulation (Fig. S1L and M). It is therefore plausible that the gregarine-like cells represent feeding and sexual stages of *C. temporaria*. Genetic recombination of *C. temporaria* within the vertebrate host would be a significant finding, as it would imply that the parasite can rapidly coevolve with host immunity and transmit directly between tadpole hosts (Fig. 9). Confirming the identity of gregarine-like cells is therefore important for reconstructing the parasite life cycle, including alternative scenarios such as spread through intermediate hosts.

Notably, the infection by *C. temporaria* in frog hosts appears to be restricted to tadpoles, whereas only empty oocysts have been observed after metamorphosis (9, personal long-term observations). This host specificity may reflect fundamental differences in immune competence between larval and adult amphibians. The apparent dependence of *C. temporaria* on macrophages is particularly intriguing because tadpoles rely predominantly on innate immunity, with Kupffer cells serving as the principal phagocytic effectors. Although these cells are capable of antigen presentation via MHC molecules, adaptive immune responses remain relatively limited during larval development (67). In contrast, metamorphosed frogs possess a much more effective adaptive immune system, in which Kupffer cells participate in antigen presentation via both MHC-I and MHC-II and contribute to the activation of helper and cytotoxic T lymphocytes (68, 69). Thus, *C. temporaria* appears to have successfully colonized a vertebrate host while remaining restricted to juvenile stages, when adaptive immunity is still immature and macrophages constitute the primary line of defense (Fig. 9).

Intracellular parasitism is rare among gregarines, particularly eugregarines, and *C. temporaria* represents the first gregarine known to infect a vertebrate host. Its intracellular infection of vertebrate macrophages is therefore unprecedented among gregarines and extends the known range of vertebrate parasitism in Apicomplexa. Moreover, to the best of our knowledge, no other apicomplexan—or indeed any other protist parasite—is currently known to selectively infect a vertebrate life stage characterized by immature adaptive immunity. This combination of vertebrate infection, intracellular development, and restriction to a developmental window of limited adaptive immunity may therefore represent a distinctive route by which gregarines could have overcome the evolutionary barrier to vertebrate parasitism.

### Conclusion

Taken together, *C. temporaria* represents a new family of Apicomplexa from freshwater hosts. It represents the fifth currently recognized independent origin of vertebrate parasitism in the group and the first among early-branching apicomplexan lineages known as the gregarines. *Cryptonaiada* was found in five frog species at a single locality, suggesting broad host range and potentially widespread presence in threatened amphibian populations. Although seemingly asymptomatic, its infection of liver macrophages, key effector cells of tadpole immunity, may affect hosts under stress. *Cryptonaiada’*s presence may therefore be relevant in the context of ongoing habitat loss, climate change, and viral, bacterial, and fungal infections, contributing to global amphibian decline over the past few decades (70). Unlike many vertebrate apicomplexans—from *Plasmodium* to *Toxoplasma* and *Babesia—Cryptonaiada* may only be able to persist in hosts dominated by a primarily innate immune response during juvenile development. However, parasites that fail to clear before metamorphosis would come into contact with adaptive immune cells; over the long term, such exposure could lead to the evolution of parasite lineages capable of evading the adaptive immune response. *Cryptonaiada* may exemplify a rare intermediate grade in parasite–vertebrate coevolution, potentially analogous to evolutionary processes that may have independently given rise to medically and economically important groups of human pathogens, such as hemosporidians and coccidians.

## Data Availability

The data underlying this study are included in this published article and its supplemental files. The RNA-seq reads generated in this study have been deposited in the European Nucleotide Archive under BioProject accession PRJEB125518 (run accessions ERR17961116 and ERR17961118). Ribosomal RNA sequences recovered from the combined assembly are available under accession numbers OZ557594 and OZ557595. The Nt12+54 transcriptome co-assembly, which contains both parasite- and host-derived sequences, is available from Zenodo at https://doi.org/10.5281/zenodo.22850965. The ZooBank registration number of the present work is urn:lsid:zoobank.org:pub:E983659F-2A58-46EC-B589-784AA87FB889. The ZooBank registration number of the new family is urn:lsid:zoobank.org:act:57AC8C28-8FED-406D-B115-2849FAEA59BB. The ZooBank registration number of the new genus is urn:lsid:zoobank.org:act:F15695D1-E1E1-4667-A163-B209782D8904.

## SUPPLEMENTAL MATERIAL

File S1 mbio.01957-26-s0001.pdf Newly assembled sequences for rRNA operon phylogeny.

Figure S1 mbio.01957-26-s0002.tif Gregarine-like cells in tadpole liver.

Figure S2 mbio.01957-26-s0003.tif Host-parasite interactions in *Cryptonaiada temporaria* oocysts.

Figure S3 mbio.01957-26-s0004.pdf ITS1 sequence relationships in *C. temporaria*.

Legends mbio.01957-26-s0005.pdf Legends for supplemental figures.

Table S1 mbio.01957-26-s0006.pdf Sequences selected for ribosomal phylogeny.

Table S2 mbio.01957-26-s0007.pdf Data sources used in the phylogenomic analysis.

ASM does not own the copyrights to Supplemental Material that may be linked to, or accessed through, an article. The authors have granted ASM a non-exclusive, world-wide license to publish the Supplemental Material files. Please contact the corresponding author directly for reuse.
