# Supplementary material for: Cellular organization and phylogeny of Cryptonaiada gen. n. (Cryptonaiadidae fam. n.) shed light on the evolution of intracellular vertebrate parasitism in Apicomplexa
Source: mBio. 2026 Oct 5;17(11):e01957-26. doi: 10.1128/mbio.01957-26 (PMC13753809; doi:10.1128/mbio.01957-26)
Supplement: Supplementary file 1 [file mbio.01957-26-s0001.pdf]

File S1. Newly assembled sequences used in rRNA operon phylogeny analysis in Figure 6.

>ex_Amphizoa_lecontei_SSU_SRR5892093
ATCTCCTGCCAGTAGTCATATGCTTGTCTCAAAGATTAAGCCATGCAAGTCTAAGTATGCGTTCAAGCACGACGAAACTG
CGAATGGCTCATTACAACAGCTATAGTCTCTGTGAAAATGATTACTACACGGATACCTGTGGTAATTCTGGAGCTAATAC
GTGCCAAAACACTGCTTGCAGTGGCACCTATCAGATTCGAACCAACCCATGCTTGCATGGAGCTCGGTGATCCAAGATAG
TCTGGTGAAGGCCTGGCAACAGGCCAAGTTCACTTGAGTTTCTGACCTATCAGCTGTCGACGGTAGGGTATTGGCCTACC
GTGGCTTTGACGGGTAACGGGGAATTAGGGTTTGATTCCGGAGAGGGAGCCTGAGAGATGGCTACCACATCTAAGGAAGG
CAGCAGGCGCGCAAATTACCCAATGCTGACACAGCGAGGTAGTGACAAGAAATATCGGTGAAAGCCATATGGTTTTCAAT
CGGAATGAGTTGAATCCAAAACTCTCTGCGAGTATCAATTGGAGGGCAAGTCTGGTGCCAGCAGCCGCGGTAATTCCAGC
TCCAATAGCGTATATTAAAGTTGTTGCAGTTGAAACGCTCGTAGTCGAATCTCAGCACGCTCAGCTGGTCTCCCTCACGG
GCTGTACTAAGCTGGGTTGCTCTGTCCTGTGTGGTGTCTGGGTAACTAGGCTCCATCAGGGTGTCACTTTGAGAAAATTT
GAGTGTTTCAAGCAGGCTTACGCCTTGAAAACTCCAGCATGGAATGACAAGTAAGGACTCCGGTTCCCCTTGTTGGTTCA
CGAACCAGAGTAATGATTAAGAGGAGCAGTTGGGGGCATTCGTACTCGGCGGCTAGAGGTGAAATTCTTAGATTCGCCGA
TGACGAACTACTGCGAAAGCATTTGCCATGGATGTTTTCATTGATCAAGAACGAAAGTGCGAGGATAGAAGAAGATCAGA
TACCTTTGTATTTCGCACCGGGACCTATACCGACTAGAAATTGGAGCGTGTCAATCATACGTCCACTCCGGCTTCTCATG
AGAAATCAAAGTCTTTGGGTTCTGGGGGGAGTATGGTCGCAAGGCTGAAACTTAAAGGAATTGACGGAAGGGCACCACCA
GGAGTGGAGCCTGCGGCTTAATTTGACTCAACACGGGAAAACTCACCAGGTCCAGACATAGGAAGGATTGACAGATTGAG
AGCTCTTTCTTGATTCTATGGGTGGTGGTGCATGGCCGTTCTTAGTTGGTGGAGTGGTCTGTCTGGTTAATTCCGATAAC
GAACGAGACATTGGCCTGCTAAATAGTCACTGGAGGTTTCCTTCAGCTGCGCTTCTTAGAGGGACTTTGCGTGTCTAACG
CAAGGAAGTTCAATGCAATAACAGGTCTGTGATGCCCTTAGATGTTCTGGGCGGCACGCGCGCTACAATGATGCGCTCAG
CGAGTTCCCCTGGCCTGAAGAGGTCGGGTAATCTTGTGAGTGCGCATCGTATCGGGGATTGACCATTGCAATTATTGGTC
ATGAACGAGGAATTCCTAGTAAGCGCAAGTCATCAGCTTGCgCTGATTACGTCCCTGCCCTTTGTACACACCGCCCGTCG
CTTCAACCGACTGGATGGTCCGGTGAATGACTCGGACTGYGAGCACYYTGGCAACAGAGAGCTTGTGGAAAGTTTTGTAA
ACCCAACCATCTGAAGGATGAAAAAGTCGTAACACGGTATCTGTAGGTGAACCTGCAGATGGATCATTCGTATTCATTCT
CACTCATTGACTCTCTC
>ex_Amphizoa_lecontei_LSU__SRR5892093
CAACGGATTGACTCCAACCATACGAATCGAAAGCTTAAACCACTCTTACATGACTTTCAGCGATGGATGTCTCGGTTCTC
GTAACGATGAAGAGCGCAGCCAGCTGCGAAATGCAGTGTGACTCGCAAACTTCAATGAATCACTAGACTCTTGAACGCAA
ACGGTACCTCTGAGCTTGCTTGGAGGTATGTTCACTTCAGCACGCTGTCTTTTAGTCTATCACTGAGGTGATTCTTGACT
GTGGGGCGCTGCTCGCATAGGGCAGAAGCTTGAAGGCATTGATCCTCTGGTGACCACCTAAGGAGAACTGCTCCCTAACC
ATGCGTCTGAAGTTGAGCAAGACTACCCGCCGAACTTAAGCATATCAGTCAGCGGAGGAAAAGAAACTAACTAGGATTCC
CTCAGTAGCGGCGAGCGAACAGGGATGAGCTCAAGTTGAAAATCGGCAGCTTGCTGACCGAATTGTAGTCTCACGAACAG
TTACCTGTCAGGTCACGGGTCTAAGTCTCTTGGAAAAGAGTACCATGGAGGGTGAAAGTCCCGTCTGTGATCCATTGATC
TGACGTTCGGTGCTGCTTCTTCGAGTCGTGTTCCTTGAGATTGGAGCACTAAACGGGTGATAAGCTTCATCCAAAGCTAA
ATACTAACGAGAATCCGATAGCATACAAGTACCGTGAGGGAAAGATGCAAAGGACTTTGAGAAGAGAGTTAAAAGGAACT
GAAATCGCTGAAAGGGAAGTGGATGGATTCGACTGCTTAGCTCGACTGGTTGTCTGACGTTAACTCGTTCGGTCGGCTTA
GTCTTGCGCTGGCTCTGGGGGCGGTTGGTGATCCTCTGTATCGCGATTCGGAAGGCACTCGTGTCGGCTGAGCGTGGGTA
AGGAGTGGTCTACTGACTGGTCATGAATGGGTGTCAAAGCCTTTACATCACCCCAGATTTGTCGGGTCTATCCATCCCGT
CTTGAAACACGGACCAAGGAGTCGTTCTCGTATGCAAGTGCGAGGGTGTTAAACCTTAGCGCGAAATCAAAGTGAGAGCT
ATGAGGGGCAACCTGCAATAGCACCCGGCTACCAGGCTGAGGTATAGCATACAGGATCGGACCCGAAAGATGGTGAACTA
TGCCTGAGTAGGGTGAAGTCAGAGGAAACTCTGATGGAAGCTCGTAGCGATACTGACGTGCAAATCGTTCGTCTAACTTG
GGTATAGGGGCGAAAGACTAATCGAACCATCTAGTAGCTGGTTCCCTCCGAAGTTTCCCTCAGGATAGCTGACATCGCCT
GGTTGCGTCAGGTAGAGCGAATGATTAGTGGAATGGGCTCTAGCTTGAGTCCACCAATTCTCAAACTTCGAATGGACGCG
AACTAGCAGTTACTTCATTGAACTGCTGGGACTAACAATGATGTCAAGTGGGCCATTTTTGGTAAGCAGAACTGGCGATG
AGGGATGCTCCTAACGTCGAGATAAGGTGCTCAAACCCCGTTCATCAGACACCATAAAAGGTGTTGGTTCATTTAGACAG
CAGGACGGTGGTCATGGAAGTTGAAATCCGCTAAGGAGTGTGTAACAACTCACCTGCCGAATGAACTAGCCCTGAAAATG
GATGGCGCTTAAACGGGGTACCGATACTCGACCATTGGCTCAAGTGTGTTGCCAATGAGTAGGAGGGCGTAGTGGTTGTG
TTGAAGTGCGTGGTGTGAACCGGCATGGAACAGCCTCTAGTGCAGATCTTGGTGGTAGTAGCACTTATTCAAATGAGAAC
TTTGAAGACTGAAGTGGAGAAGGGTTCCATGTGAACAGCAATTGGACATGGGTCAGCCGGTCCTAAGGGGGAGATTCGTC
TCTACTATGCTAGCTTCGGCTACGCACTCCGAAAGGGAATCGGGTTAACATTCCCGAGCCTGGGCGTGGATATTGTGTGG
TAACACAAAGGAACTTGGGTCCGTAGGCCATTGCTCCGCACAGCGTTGTCTTTGCTGTCTAACAGTGTGATACCCCTGGA
ATTGGATTGTCCAGAGATAGGGGTCTCAAGCTGGAATAGGATTCCAATCTTCGGAGTCCGCGGCTCGCAGTGTCTTACCC
CTGAAACCCCAAGGGACAATCTTATTCGCACCCCTGGCCGTACCAACAACCGCATCAGGTCTCCAAGGTTCGCAGCCTCT
GGTCAGTAGACTAAAGTAGCTAAGGGAAGTCGGCAAAATAGATCCGTAACTTTGGGAAAAGGATTGGCTCTGAGAGCTGG
GCTTGGGGACTCCTGGACTTAAGCCTTGGTCGGGTTTGAGCCTATCTGAAGCCTTCGGGTGGATGATGGACTCATTCAAG

GTCGGGTAGCGGCTCTTGAGTGCCCTTGCGAGAAGCAGCTAACTCAGAACTGGAGCGGACAAGGGGAATCCGACTGTTTA
ATTAAAACAAAGCATTGCGATGGCCTCGGATGGTGTTGACGCAATGTGATTTCTGCCCAGTGCTCTGAATGTCAACGTGG
TGAAAATCAAGCAAGCGCGGGTAAACGGCGGGAGTAACTATGACTCTCTTAAGGTAGCCAAATGCCTCGTCATCTAGTTA
GTGACGCGCATGAATGGATTAACGAGATTCCTACTGTCCCTAGCTACTATCTAGCGAAACCACAGCCAAGGGAACGGGCT
TGGAAAAATCAGCGGGGAAAGAAGACCCTGTTGAGCTTGACTCTAGTTTATCGCTGTGGAAGGACTCAGGGGGTGTAGCA
TAAGTGGGAGACCCGTCAACCTTGAAATACCACTACCCTTGACGTCCTTCTACTTACTCCATGACGCTGAGGCTCGCTTA
GGTGAGTTCTTGCGTTAACGCAGCTTGCTGCTGATCAGAGTGGATGACACCGATAGGTGGGGAGTTTGGCTGGGGCGGTA
CATCTGTTAAACAACAACGCAGGTGTCCTAAGGCGAGCTCAATGAGAACAGAAATCTCATGTTGACCAAAAGGGGAAAAG
CTCGCTTGATTTTGACTTTCAGTACGAGTACAAACCGCGAAAGCGTGGCCTATCGATCCTTTAGATCTGAGAAGCTTTCA
GCTAGAGGTGCCAGAAAAGTTACCACAGGGATAACTGGCTTGTGGCAGCCAAGCGTTCATAGCGACGTTGCTTTTTGATC
CTTCGATGTCGGCTCTTCCTATCATTGAGGACGCAGAAGTCCCAAAGTGTCGGATTGTTCACCCGCTAATAGGGAACGTG
AGCTGGGWTTAGACCGTCGCGAGACAGGTTAGTTTTATCCTACTGATGACTGCTCTGCCTTAGTAACCCAACTTAGTACG
AGAGGAACAGTTGGATCGGATACTTGGTAATGCAGCTGGTTGAAAAGCCAGTGCTGCGAAGCTATCACCCGCTCGGTAAA
GACTGAACGCCTCTAAGTCCGAACCGATGCTAATGGCAGKGCTATTCRTYGCTCTCTAGTACATCTGTCAACAGATACTT
ATCCAATGCTCGATCAATCGATATCCTTGACTTGAGAGTGGTACATCCTGTGAGGACGACGCAGATGGCAGCAGGCATTG
TAAGCATGAGAGCAGACTTTGTTCTGCGATCTGCTGAGATCTAGCCTGGCTGTC
>ex_Hydrochara_caraboides_LSU_partial__SRR2083674
CTCAGTAGCGGCGAGCGAACAGGGATGAGCTCAAGTTGAAAATCGGCAGCTTGCTGACCGAATTGTAGTCTCACGAACAG
CTACCTGTCAGATTACGGGTCTAAGTCTCTTGGAAAAGAGTACCATGGAGGGTGAAAGTCCCGTCTGTGATCCATTGATC
TGACGTTCGGTGCTGCTTCTTCGAGTCGTGTTCCTTGAGATTGGAGCACTAAACGGGTGATAAGCTTCATCCAAAGCTAA
ATACTAACGAGAATCCGATAGCATACAAGTACCGTGAGGGAAAGATGCAAAGGACTTTGAGAAGAGAGTTAAAAGGAAC
>ex_Hydrochara_caraboides_LSU_partial_scaff_Middle__SRR2083674
GGAGTCGTTCTCGTATGCAAGTGCGAGGGTGTCAAACCTTAGCGCGAAATCAAAGTGAGAGCTATGAGGGGCAACCTGCA
ATAGCACCCGGCTACCAGGCTGAGGTATAGCATACAGGATCGGACCCGAAAGATGGTGAACTATGCCTGAGTAGGGTGAA
GTCAGAGGAAACTCTGATGGAAGCTCGTAGCGATACTGACGTGCAAATCGTTCGTCTAACTTGGGTATAGGGGCGAAAGA
CTAATCGAACCATCTAGTAGCTGGTTCCCTCCGAAGTTTCCCTCAGGATAGCTGACATCGCCTGGTTGCGTCAGGTAGAG
CGAATGATTAGTGGAATGGGCTCTAGCTTGAGTCCACCAATTCTCAAACTTCGAATGGACGCGAACTAGCAGTTACTTCA
TTGAACTGCTGGGACTAACAATGATGTCAAGTGGGCCATTTTTGGTAAGCAGAACTGGCGATGAGGGATGCTCCTAACGT
CGAGATAAGGTGCTCAAACCCCGTTCATCAGACACCATAAAAGGTGTTGGTTCATTTAGACAGCANNNNNNNNGTCATGG
AAGTTGAAATCCGCTAAGGAGTGTGTAACAACTCACCTGCCGAATGAACTAGCCCTGAAAATGGATGGCGCTTAAACGGG
GTACCAATACTCGACCATTGGCTCAAGTGTGTTGCCAATGAGTAGGAGGGCGTAGTGGTTGTGTTGAAGTGCGTGGTGTG
AACCGGCATGGAACAGCCTCTAGTGCAGATCTTGGTGGTAGTAGCACTTATTCAAATGAGAACTTTGAAGACTGAAGTGG
AGAAGGGTTCCATGTGAACAGCAATTGGACATGGGTCAGCCGGTCCTAAGGGGGAGATTCGTCTCTACTATGCTAGCTTC
GGCTACGCACTCCGAAAGGGAATCGGGTTAACATTCCCGAGCCTGGGCGTGGATATTGTGTGGCAACACCAAGGAACTTG
GGTCCGCAGGCCATTGCTCCGCACAGCGTTGTCTTTGCTGTCTAACAGTGTGATACCCCTGGAATTGGATTGTCCAGAGA
TAGGGGTCTCAAGCTGGAATAGGATTCCAATCTTCGGAGTCCGCGGCTCGCAGTGTCTTGCCCTTGAAACCCCAAGGGAC
AATCTTATTCGCACCCCTGGCCGTACCAACAACCGCATCAGGTCTCCAAGGTTCGCAGCCTCTGGTCAGTAGACTAAAGT
AGCTAAGGGAAGTCGGCAAAATAGATCCGTAACTTTGGGAAAAGGATTGGCTCTGAGAGCTGGGCTTGGGGACTCCTGGA
CTTAAGCCTTGGTCGGGTTTGAGCCTATCTGAAGCCTTCGGGTGGACGATGGACTCATTCAAGGTCGGGTAGCGGCCCTT
GAGTGCCCTTGCGAGAAGCAGCTAACTCAGAACTGGAGCGGACAAGGGGAATCCGACTGTTTAATTAAAACAAAGCATTG
CGATGGCCTCGGATGGTGTTGACGCAATGTGATTTCTGCCCAGTGCTCTGAATGTCAACGTGGTGAAAATCAAGCAAGCG
CGGGTAAACGGCGGGAGTAACTATGACTCTCTTAAGGTAGCCAAATGCCTCGTCATCTAGTTAGTGACGCGCATGAATGG
ATTAACGAGATTCCTACTGTCCCTAGCTACTATCTAGCGAAACCACAGCCAAGGGAACGGGCTTGGAAAAATCAGCGGGG
AAAGAAGACCCTGTTGAGCTTGACTCTAGTTTATCGCTGTGGAAGGACTCAGGGGGTGTAGCATAAGTGGGAGATCCGTC
AACCTTGAAATACCACTACCCTTGACGTCCTTCTACTTACTCCATGACGCTGAGGCTCGCTTAGGTGAGTTCTTGCGTTA
AAGCAGTTTACTGCCGATCAGAGTGGATGACACCGATAGGTGGGGAGTTTGGCTGGGGCGGTACATCTGTTAAACAACAA
CGCAGGTGTCCTAAGGCGAGCTCAATGAGAACAGAAATCTCATGTTGACCAAAAGGGGAAAAGCTCGCTTGATTTTGACT
TTCAGTACGAGTACAAACCGCGAAAGCGTGGCCTATCGATCCTTTAGATCTGAGAAGCTTTCAGCTAGAGGTGCCAGAAA
AGTTACCACAGGGATAACTGGCTTGTGGCAGCCAAGCGTTCATAGCGACGTTGCTTTTTGATCCTTCGATGTCGGCtCTT
CCTATCATTGAGGACGCAGAAGTCCCAAAGTGTCGGATTGTTCACCCGCTAATAGGGAACGTGAGCTGGGTTTAGACCGT
CGCGAGACAGGTTAGTTTTATCCTACTGATGACTGCTCTGCCTTAGTAACCCAACTTAGTACGAGAGGAACAGTTGGATC
GGATACTTGGTAATGCAGCTGGTTGAAAAGCCAGTGCTGCGAAGCTATCACCCGCTCGGTAAAGACTGAACGCCTCTAAG
TCCGAACCGATGCTAATGGCAGGGCTATTCGTCGCTCTCTAGTACATCTGTCAACAGATACCTTTCCAATGCTCGATCAA
TCGATATCCTTGACTTGAGAGTGGTACATCCTGTGAGGACGACGCAGATGGCAGCAGGC

>ex_Hydrochara_caraboides_58S__SRR2083674
CTCAGTAGCGGCGAGCGAACAGGGATGAGCTCAAGTTGAAAATCGGCAGCTTGCTGACCGAATTGTAGTCTCACGAACAG
CTACCTGTCAGATTACGGGTCTAAGTCTCTTGGAAAAGAGTACCATGGAGGGTGAAAGTCCCGTCTGTGATCCATTGATC
TGACGTTCGGTGCTGCTTCTTCGAGTCGTGTTCCTTGAGATTGGAGCACTAAACGGGTGATAAGCTTCATCCAAAGCTAA
ATACTAACGAGAATCCGATAGCATACAAGTACCGTGAGGGAAAGATGCAAAGGACTTTGAGAAGAGAGTTAAAAGGAAC
>ex_Mytilus_californianus_SSU_scaff__SRR5275489
ATGCTTGTCTCAAAGATTAAGCCATGCATGCGAAAGTATAAGACTTTATACGTCGAAACCGCAGACGGCTCATTAAAACA
GTTATGATCTACACGCATATTGATTACACGGCTAACCGTGGTAATTCTGGGGATAATACGTGCAAGCTTCGGCTTACTCT
CTTTCAGAGTTGTTGTAGAAAACAGCATTCGCACTATCACCATTTGAATAAGTCTACAATTCAATTGATTTCAATGATGC
GTTTGAATATCTGATCTATCAGTTCTGACGGTAGTGTAGTGGACTACCGTGACTGTAACGGATAACGGAGAATTAGGGTT
CGATTCCGGAGAAGGAGCCTTAAAAACAGCTACTACATCTAAGGAAGGCAGCAGGCGCGCAAATTGCTCAATGAAGGTCA
TTCGAAGCAGTGACAAGAAATATCAAAGCCAGCTTTCAGCTCGCTATTGATCTGAGGGTAATTTAAAAACTTACTCGATT
ATTATTGGATCGCTAGTGGGGTGCCAGCCGGNNNNNNNNNNNNNNNNCCAATAGTGTATGCTAAAATTGTTGCAGTTAAA
ACGCTCGTAGTCGTAGTTTCTTGTTTCTTTCAGCACCATTCGGAGCTGTTAGTAACTTTACTGAAACATTCGTTTCATAT
TACCGTGAATAAAGGGGAATGCTCCAAGTAAGCTGTTGCTTTGAACCTTTAAGCATGGGATAATGAACACGGCGGTTGCC
ACCTCGTGAATGGGTGGTTTCTGCTTTCTAGCAGGATTGAAGAGGGACACTCGTACTCAACAGCCAGAGGTGAAATTCTT
AGACTTGTTGAAGACGAACAGATGCGAAAGCGATTGTCAGATTCGAATCCAGTGATCAAGAACGAAATTCAAGGGATCGA
AGATGATTAGATACCGTCGTAGTCTTGGACATAAACTATGTCGACTAACACTTGGAGATGAAAATCAATACCATCTTCAG
GGGTTACTGGGAAACCAAAGTCTTCGGATTCTGGGGGGAGTACATGCGCAAGCACGAAACTTAAAGAGATTGACGGAAGC
GTACCACCAGAAGTGGATCGTGCGGATTAATTTGACYCAACACGGAGAATCTTACCAAGTTCATACATTAGAATGATTGT
CAAGGTAGAATGGTTTTTACTTGATTTAATGGGAGGTGGTGCATGGCCAAACTTAGTTCGTGGATTGATCTGTCTGGTTT
ATTCCGTTAACGAACGAGATCTCGACTTTTAATTACGGCACAATGGTATTCTCCATCGCTGTCGATTTTAAAAGGACACT
CTGTTTAAAACAGCAGGGAGGTTCGAGGTTAATTTAGGTCTGTTATGCCCTTCGATAACTTGGGCCTCACGCGCGATACA
ATGCAAGATTCTATTTCTCTCTTATTAGTAATGATAAGAAAATTCTCAATACCTTGCCTGACTGGGATTGTTCATTGAAA
TTTTTGATCATGAACGAGGAATTTCTTGTAAGCATGGAGTCATCAACTTCTGCTGAATATGTCCCTGTGCTTTGTACACA
CCGCCCGTCGTTTCATCCGACTGGAAGTTATGGTGAATGATCTGGATCGATTGCGCAAGCATCGAAAAGGATTGTAAATC
TTAACGTCTGAAGGATGAA
>ex_Mytilus_californianus_LSU_cont_1__SRR5275489
ACATTCCTTGGGCTACCCACCGAACGTAAGCATATTACTATGTGGAGGAAAAGAAAATAAAAATGATCCCTTTAATAGTG
GCGAACGAAACAGGGACAAGATTAAGGGCGCCATCACTCCTCCCCTCGGGAGGGTGTAAATGCCTTGGGTGCGCATGAGA
CTGCGGTTGCTCTTCAGCTTTTGAGATATTCGACGAATACGATATAATGTATCTGTAAGTCTGCAACCAACGTCTGTCAC
ACGCGTATCCCCATAAGTCGGACTTATTGCGAGTTAAGTCCAATTGGAGATGATCGTCTTCGAGATCTGAATATAATCCT
ATACCGATAGCAAACAAGTACCATGAGGGAAAGTTGAAAAGCACTCATAAAAGAGAATTAATTGAACTTGAAATCGATAT
GGTAGAACGGTATTTCAGGAGCACGACATGTTCTGAAGTTGCTTTCGCTTCAACCTTCAGTTCTGTCACCATGTTCTCAT
GTCTCGAAGGGAGACGGCAGTATTCGAACTTCGGTTTGCATTCTGTCTCTCTGTTAAGAGGTATCCTGCCGATTTATGTG
AACATGCTTCCCGATTTTACCACCCGTCTTGTAACACGGACCAAGGAGTGTTAGGATGTTGCGGGGTGTTTCATTGAATT
GAGTAGCACTCGTGTCTGAGCAAGACTGACTCTCTTTTTCAAGATCATTAGCAGTATTCTAACGACCCGAAAATCGGTGA
ACTTTCCCTGTGAAGGGTGAAGCCAGAAGAAATTTTGGTGGAAGCCCGGAACTGTGCTGACGTGCAAATCGCTTGTCTTA
CATGGGGAAAGGGGCGAAAGACCAATCGAACCGATTAATAGCTGGTTACCTCCGAAGTTTCCCTCAGGAAAGGTGCGTCT
CGTTCCCTAAAGCAGGGACGGTAAAGCTTATGATTAGAAGTACCGGGGGAGAATTCCCTCGACTGATTCTCAAACTTTAA
ATGTTCCTTAGTCGTCATCCTCACGGAATGCGACTGGTGCTTAGAGATGTTACTGGGCCTGTATTTGTAAGAAATACCGG
CGATGTGGGATAATCCGCAAGTCAAGTGAACCCGCTAAAGTGTAAGCTGGGTAAAATCAAAAGGTGTTTCTATATTTAGA
CAGCAGGACGGTGGCCCTGGTAGTCGGAACCCGCTAAGGAGTGTGTAACAACTCACCTGCCGAATGTAGTTACCCTGAAA
ATGGATTGCGCTAAAGCTTACAGGGGATACTTGACCTTCTCTAAATCTAGAGAAGTGTAGGAGGTTGTAAAGGAGACATG
TGATTGCCAGTGATGGCATCGTTCAT
>ex_Mytilus_californianus_LSU_cont_2__SRR5275489
CGTGGTCCGATGTGAGGTAGGTTTTCACGGCAACTTCATGTGGCCGTGAGTGAGACGATCCTAAGCGGTGCATTAATCTG
TTTCATCCCTGATTCCAGGTGTTCACTGCGAAAGGGAAACGAGCGTTGAATCTCGTTCCAATATCCTCCAAAGGGCGACA
TGATTGTGCTACGCCTCGTTTTGACTATGAACCTTATGCGTTTCCGGCGCATATCAACAAACTGAACACTGTTTAATTGG
TGCAACCAGAGAGACAGTGAGCATTTGGATTAGGTTGCTCTTCATGAGCGGTCTACGTTTCATGTCAGAACCCTTGAAAA
GCTAAGCACAGTATGGAGTTGATTGATCGTACCCATATCCGCATCAGGTCCTCAAGGTTAAATAGCCTCTGGTTCCTAGA
ATATCGTAGGTAAGGGAAGTCGGCAAATTGATTCCGTACCTTAGGAAAAAGGAATGGCTCTGAAGGTTTGTGATTCTCTG
TGTAGAGGACTAGCGATGTTTGTATATGTGGCACCTTCGGGTCTACTAACATTCACGCGGGGCCTCTTTCATGGCGTTTC
ACGCTAACAACCTTTTCAGAACTGCAGCGGACAAGAGGAATCCGACAGTTTAACTAAAACCAAGCTTTTCGCAAACGTTG

AGACGTTTATATGAAAGGTGATTTCTGCCCAATGCGCTGTTTGTCAAGTTGTAGAATTTAGACACAATTAAGCGCGCGTC
AATGGCAGGGGTAACTGTGACCCTTTTAAGGTAGCCAAATGCCTCGTCATCTAATTAGTGACGCGCATGAATGGATCAAC
GAGACTCCTACTGTCCCTATCTGCGTTCTAGTGAAACCACAGCCAAGGGAACGGGCTTGGCTGTTATATAGACAGCGGGG
AAAGAAGACCCGGTTGCGCTTGACTCTATTTTGGGATTGTAAGAGGACTTGAGAGGTGTAGCATACGTGGGAGTCTTCGG
ACGTCCTTGAAATACCACGACTCTCAATGTCTTTTTACTTATCTCTTGACCAATCACTTCGTTCTGCGATCTATGTGAGA
GAGAGACATTCTCAGATGGGGAGTTTGGCTGGGGCGGTACATCTGTTATAACAAACCACAGGTGTCCTAAGGCAAAGTCA
TGGAGAACAGAAATCTCCAGTTGACCAAAAAGGGAAAACTTTGCTCTACTGGTTTGTTCAGCACACAAATCAGTTCTGAA
AGGAAGGCCTATCGATCCTTAGCGCATTTTTTTCCAACGATCGGACGCTGAGGTGCCGGAAAAGTCACCACCGGGATAAC
TAGTTCGTGGCCGCCGAGAGATCATATCGACGCGGCAAGTTGATTTTTCGATGTCGGCTCTTTCTATCCTAAGCGTGTAG
AAGCGCTTAATGGTCGGATTGTTCACCCGTAGTTAAAGGGAACGTGAGCTGGGTTCAGACCGTCGTGAGACAGGTTAGTT
TTATCCTACTGTCTTCTTCCTTCTAAAGGATACTAGTCAAGTACGAGAGGAACGACTAGTACGCACCTACAGCTCTGCGG
CAGGAGGGAACTCCAAGGCTGCAAGACCGCGTGCGCAGGATAAATATTGAATGCCTCTAAAACTGAAACCTCTCTTTGCG
GAGGATTACACTCCTTATTGACAGATCATTCTTAACAATCTTCAAAGGAGTAGACCGAATGAAAGACCATCATGCAAAAG
TGTAAGTTGTACGGTTTAAGCACCTTTATGATGCGATTT
>ex_Cerastoderma_edule_ITS_LSU_part1__SRR14870539__SRR14870540__SRR14870542
ACTTACCCTGTAAAGGGTCTCAAGTCCACTTGTGGACTAACACCTTTGATTTGAAAGAAGTTTGTTCTACTGTCAAATCT
ATGGTAATTAAAGTATCGATGAAAGTGTTGGATAGGATAACGATGAAGGGCGCAATGTACCGCGAAAATTCGAATCTACT
GAATTACATCCCTTAAAAGAAAATACCTCAAAATCTACGTGCCCTTTCCAGGGCATTTGGAAATGTCAGACATTGATAGG
TGTTGAACTACCGCCTTTGGCCAATTTTACTGCCATATGCTGTTGAAAACAACTCTTTGTTTACCATCCTACAATAACTA
ATGATGACATTTCTTGGGCTACCCACCGAACGTAAGCATATTACTATGTGGAGGAAAAGAAAATAAAAATGATCCCTTTA
ATAGTGGCGAACGAAACAGGGACAAGATTAAGGGCGACATCATTCCTCCTTTTAGGAGGGTGTAAATGCCTTGGGTGCGC
ATGGGACCGCGGTCGCTCTTCAGCTTTTGAGATATTCGACGAATACGATATAATGTATCTGTAAGTCTGCGACCAACGTC
TATCACACGCGTATCCCCATAAGTCGGACTTATTGCGAGTTAAGTCCAATTGGAGATGATCGTCTTCGAGATCTGAATAT
AATCCTATACCGATAGCAAACAAGTACCATGAGGGAAAGTTGAAAAGCACTCATAAAAGAGAATTAATTGAACTTGAAAT
CGATATGGAAGAACGGTATTTCAGGAGCACGACATGTTCTGAAGTTGCTTTCGCTTCAATCTTCAGTTCTGTCATCATGT
TAGCATGTCTCGAAGGGAGTTGGTTAGTAGCAATACTTCTGTATTGCAAACGATCTCTCTGTTAAGAGGTATCCTGCCGA
TTTATGTTAACATGCTTCCCGATTTTACCACCCGTCTTGWAACACGGACCAAGGAGTGTTAGGATGTTGCGGGTTGTTTC
ATTGAAATGAGTAGCAATCGTGTCTGAGCAAGACTGACTCTCTTTTTTAAGATCATTAGCAATATTCTAACGACCCGAAA
ATCGGTGAACTTTTCCTGTGAAGGGTGAAGCCAGAAGAAATTTTGGTGGAAGCCCGGAACTGTGCTGACGTGCAAATCGC
TTGTCTTACATGGGAAAAGGGGCGAAAGACCAATCGAACCGATTAATAGCTGGTTACCTCCGAAGTTTCCCTCAGGAAAG
GTGCGTCTCGTTCCCTAAAGCAGGGACGGTAAAGCTTATGATTAGAAGTAACGGGGGAGAATTCCCTCGACTGATTCTCA
AACTTTAAATGTTCCTTAGTCGAAATCCTCACGGAATTAGACTGTTGCTTGGAGATGTTACTGGGCCTATATTTGTAAGA
AATATCGGCGATGTGGGATAAACCGCAAGTCAAGTGAACCCGCTAAAGTGTAAGCTCGGTAAATTCAAAAGGTGTTTCTA
TATTTAGACAGCAGGACGGTGGCCCTGGTAGTCGGAATCCGCTAAGGAGTGTGTCACAACTCACCTGCCGAATGTAGTTA
CCCTGAAAATGGATTGCGCTAAAGCTTACAGGGGATACTTGACCTTCTCTAGATTTAGAGAAGTGTAGGAGGTTGTAAAG
GAGACTTGTGATTGCCAGTGATGGCATCGTTTATCTCCAATTAGTGCAGATCTTGGTGGC
>ex_Cerastoderma_edule_part2__SRR14870539__SRR14870540__SRR14870542
TCTGTGAGGTAGGCTTTCACGGCAACTTCATGTGGCCGTGAGTGAGACGATCCTAAGCGGCGCATTAATCTGTTTCATCC
CTGATTCTAGGTGTTTGCTGCGAAAGGGAAACGAGCGTTGAATCTCGTTCCAATATCCTCCAAAGGGCGACATAATTGTG
CTACGCTTCGTTTTGACTATGAACCTTATGCGTTTCCGGCGCATATCAACAGATTGAACACTGTTTAAGTGGTGCAACCA
GAGAGACAGTGTACATTTGGATTAGGTTGCTCTCTGTGAGCGGTCTACGTTTCATGTCAGAACCCTTGAAAAGCTAAGCA
CAGCATGGAGTTGATTGATCGT
>ex_Cerastoderma_edule_part3_SRR14870539_SRR14870540_SRR14870542
CTTCCTTCTAAAGGATACTAGTCAAGTACGAGAGGAACGACTAGTACGCACCTACAGCTCTGCGGCAGGAGGGAACTCCA
AGGCTGCAAGACCGCGTGCGCAGGATAAATATTGAATGCCTCTAAAACTGAAACCTCTCTTTGCGGAGGATTACACTCCT
TATTGACAGATCATTTTTAACAATCTTCAAAGGAGTAGACCGAATGAAAGACCATCATGTAAAAGTGTAAGTTGTACGGT
TTAAGCACCTTTATGATGCGATTTCCTAAGACTCTGCACT
>ex_Litopenaeus_vannamei_SSU_SRR6466326
CATACATCTAAACCTATTCGACTCATTTGAGCTGAATAAAGATGTAACCCGGTTGACTCTGCCGGTAGTATATGCTTGTC
TCAAGGATTAAGCCATGCATGCGAAAGTATAAGACTTTATACGTCGAAACCGCAGACGGCTCATTAAAACAGTTATGATC
TACACGCATTTTGATTACACGGCTAACCGTGGTAATTCTGGGGATAATACGTGCAAGTTTCGGCTTCACTCTCTTTCGGA
GTTGTTGTAGAAAACCTCGCATCCGCACCTTCACCATTTGAATAAGTCTACAATTCAATTGACTCGTCAATGATGCGTTT
GAATATCTGATCTATCAGTTTTGACGGTAGTGTAGTGGACTACCGTGACTGTAACGGATAACGGAGAATTAGGGTTCGAT
TCCGGAGAAGGAGCCTTAAAAACAGCTACTACATCTAAGGAAGGCAGCAGGCGCGCAAATTGCTCAATGAAGGTCATTCG

AAGCAGTGACAAGAAATATCAAAGCCAGCTTTTAGCTCGCTATTGATCTGAGAATGATTTAAAAACTTACTCGATTATTA
TTGGATCGCTAGTGGGGTGCCAGCCGGAGCGGTAATACCTCCTCCAATAGTGTATGCTAAAATTGTTGCAGTTAAAACGC
TCGTAGTCGTAGTTTCTTGGCACTTTGGTTGACTACCAACATGGAGTCGCTAGTGTCTTTACTGAAACACTCGTGTTTCA
TATTACCGTGAATAAAGGGAATTGCTCCAAGAAAGCTGTTGCTATGAACCTTTAAGCATGGGATAATGAACATGGCGGCT
GCTACCATCGTGTGGATGGGTAGTTGCTGCTTTCTAGCAGGATTGAAGAGGGACACTCGTACTCAACAGCCAGAGGTGAA
ATTCTTAGACTTGTTGAAGACGAACAGATGCGAAAGCGATTGTCAGATTCGAATCCAGTGATCAAGAACGAAATTCAAGG
GATCGAAGATGATTAGATACCGTCGTAGTCTTGGACTTAAACTATGTCGACTACCACTTGGAGACGTTCACAAACAACGC
CTTCAGGGGGTCCTGGGAAACCAAAGTCTTCGGATTCTGGGGGGAGTACATGCGCAAGCACGAAACTTAAAGAGATTGAC
GGAAGCGTACCACCAGAAGTGGATCGTGCGGATTAATTTGACTCAACACGGAGAATCTTACCAAGTTCATACATTAGAAT
GATTGTCAAGGTAGAATGGTTTTTACTTAATTTAATGGGAGGTGGTGCATGGCCAAACTTAGTTCGTGGATTGATCTGTC
TGGTTTATTCCGTTAACGAACGAGATCTCGACTTCTAATTACGTCTCAGTATATTAAAATACTGGTCTACGCTTTAGAAG
GACACTCTGTTTAAATCAGCAGGGAGGTTCGAGGTTAATTTAGGTCTGTTATGCCCTTCGATAACTTGGGCCTCACGCGC
GATACAATGCAAGATTCTATTTCTCTCTAGTTAGTAATGATTAGAAAATTCTCAATACCTTGCCTGACTGGGATTGTTCA
TTGAAATTTTTGATCATGAACGAGGAATTTCTTGTAAGCATGGAGTCATCAACTTCTGCTGAATATGTCCCTGTGCTTTG
TACACACCGCCCGTCGTTTCATCCGACTGGAAGTTATGGTGAATGATTCGGATCGCTTGCGCAAGCATCGAGAAGGATTG
TAAATCTTAACGTCTGAAGGATGAAAAAGTCGTAACACGGCAACAGTTGTTGAAACAGTTGTTGGATCATTCCTGAACTG
TCTTTCGGCATCCG
>Polyplicarium_lacrimae_SSU__SRR21755685
CCTGCCAGTAGTCATATGCTTGTCTCAAAGTTAAGCCATGCATGTCTAAGTATAAGTACTCGACTGACGAAACTGCGAAC
GGCTCATTAAAACAGTAATAATTGGTGTGAAGGGCAGTCATACACGGGCAACCGTGGGAAACCAAGGCTAATACGTGCGA
ATGGCCTCGGCCAGCGTTTATTAGATAAAGAGCCAGCAATCGGTGTTTCATGGTAACCAATCTGATCGCACGCTTCGGCG
GCGACGGTTCATTCAAGTTTCTGACCTATCAGCTCTCGACGGTCGGGTATTGGCCCACCGTGGCGTTAACGGGTGACGGG
GAATTGGGGTTCGATTCCGGAGAGGGAGCCTGAGAGATGGCTACCACGTCTAAGGATGGCAGCAGGCGCGCAAATTACCC
AATCCAGATTCTGGGAGGTAGTGACAAGAAATAGCAGGTCGAGACATACGTCTTGGGATTGCAATGAGTAGAATCTAAAC
GTTTCTACGATTATCAATTGGAGGGCAAGTCTGGTGCCAGCAGCCGCGGTAATTCCAGCTCCAATAGCGTATATTAAAAT
TGTTGCAGTTAAAACGCTCGTAGTTGAATGTCTGTCTCGTGGTGTTCCGGTCCGCTTTCAGCGTGTACTTGGCACCAGTG
GCATCCGTTTGTCGGCACTAGTGTCGCACAAGCCATTCACTTTGAGGAAATTAGAGTGTTCAACGCAGGCTGTTGTCTTG
AATACTTCAGCATGGAATGATCGATAGGACTTCGGCCCTGTCAGTTGGTTCGAGGGGTGAGGAGTAATGATGAAAAGGGT
CGGTCGGGGGCATTCGTATTGAACAGTCAGAGGTGAAATTCTAAGATTTGTTCAAGACGCGCTACTGCGAAAGCATTTGC
CAAGTATGACTCCATTAATCAAGAACGAAAGTTAGGGGATCGAAGACGATCAGATACCGTCGTAGTCTTAACCATAAACT
ATGCCGACTAGAGATTGGGGGCTGTCTTTTGACTCCTTCAGCATCTTCAGAGAAATCCAAGTTTTTGGGTTCTGGGGGGA
GTATGGTCGCAAGGCTGAAACTTAAAGGAATTGACGGAAGGGCACCACCAGGAGTGGAGCCTGCGGCTTAATTTGACTCA
ACACGGGGAAACTCACCAGGTCCAGACGTAGGCAGGATTGACAGATTGATAGCTCTTTCTTGATTCTATGGGTGGTGGTG
CATGGCCGTTCCTAGTTCGTGGATTGATTTGTCTGGTTGATTCCGTTAACGAGCGAGATCTTAGCCTGCTAACTAGGTCG
ACAGAGCATGAGGTCTGTTCGTACCTTCTTAGAGGGACATTGCGCGTTTAGCGCACGGAAGTTTGAGGCAATAACAGGTC
TGTGATGCCCTTAGATGTTCTGGGCTGCACGCGCGCTACACTGACACTTTCAGCAAGTGCCCTGCTCCGTGAGGACTGGG
TAACCAACTGAGTCTGTGTCGTGATGGGGATAGAATATTGTAATTATTACTCTTGAACGAGGAATTCCTAGTAAGCGCGA
GTCAGCAGCTCGCGCTGATTACGTCCCTGCCCTTTGTACACACCGCCCGTCGCTCCTACCGATTGGGTGATCCGGTGAAT
TATTCGGACTCTGATGGTGCTAGCACATGGTGCTATTGGCGAAAGTTTTGTGAACCTTATCACTTGGAGGAAGGAGAAGT
CGTAACAAGGTTTCCGTAGGTGAACCTGCGGAAGGATCATTCGCGGTTCTGGTTGGCTGGCATTGGCTGATTGACAGAGA
GAGAGACAGGTTG
>Polyplicarium_lacrimae_LSU__SRR21755685
ACATGGTGCTATTGGCGAAAGTTTTGTGAACCTTATCACTTGGAGGAAGGAGAAGTCGTAACAAGGTTTCCGTAGGTGAA
CCTGCGGAAGGATCATTCGCGGTTCTGGTTGGCTGGCACATTGGCTGTTTGACAGAGAGAGAGACAGGTTGATCGTGGTG
GACCCCCCTTATATTTACTGGCAGGAGTTGTTGATTGTTTTATAATGAACTTCGGTGATGGATGGGTCGGCTCTTGTATC
GATGACGGCCGCAGCAAGCTGCGATACGCAATACTAATTGCAGAAATCATGATTTACTAGGTCGCTTAACGCAATTTGTG
TGACTGCTTGCCAGTCGCAGGTTCTCCTCAGTTTGGTAATCTAATTCTTTGGTTTGACAGGTGTAAGGACCTGCAAATCC
GTCATTTACGCCGTTGCATGGCACGAGAGCGTGCAGGCGTCTGGTAATTCAACTGAGTGGAGCCAGTATACCCGCTGAAT
TTAAGCATATAACTCAAGCGGAGGAGGAGAAACCAAATGGGAATCCCCTAGTAGCGGCGAGCGAACAGGGACCAGCTCAC
AATGCAAACTCTTAACGAGGTTGTGGTGTTGTGAAGTGTCACCGGCTTTACGCAGCTGGCCAGTCTTCTGAAAAGAGGTA
CCGTGGGAGGTGATAGTCCTACGCCAGCTTACCGTCCTGCGTGTGGTGCACTTTCGCAGAGTCGTGCTCCTTGGGATTGG
AGTGCTAATTGGGTGGTAAATTTCATCCAAAGCTAATTATATGCAAGAGACCGATAGACGACAAGTACCGTGAGGGAAAC
GTGAAAAGAACTTTGGAAAGAGAGTTAAATAGTGCCTGAAATTGCCGAAGAAGAAGTGAGTTATGGTCACAGGAACAGAG
GGGATTGGCCCCTCTGCTTTGGACTCATGGTAGTTGTTGCCGTATCTTCTTCGTGTGTTGACTGCACAAGTGTGGTCTTC

CCGGAGTCCCTACTCCAGCTTCTGCTCGCTGTTAGCAGCGTCGGTCCTGGTGACTTGACCCCTTCCCGTCTTGTAACACG
GACCAAGGAGTCTAACATGTTTGCGAGTGTGAGGGTTTTTAAACTCTCGTGCGAAGTGAGAGCGAGTTGTTTSTTTTGAG
CAGGCCGGCCTTGTTCCTTGGAACTTGGTTGCGTCAGAGCAAACCTGTTAGGACCCGAAAGGTGGTGAACTATTCCTGAG
CAGGCTGAAGCCAGGGGAAACCCTGGTGGAAGGTCGTCGCGATACTGACGTGCAAATCGTTCGCTAAACTTKGGTATAGG
GGCGAAAGACTCATCGAACCACCTAGTAGCTGGTTCCCCTCGAAGTTTCCCTTAGGATAGCTGGTGATCGTTGGTGGGCA
GTTTTGTCAGGTAAAGCGAATGATTAGAGGCATTGGGGGTGTGTCACCCTCGACCTATTCTCAAACTTTGAATCGGCAAG
AGAGACTGTTGCTTCTATGAACAGTCGTGTGAATGTGATCGCCAAGTGGGCCATTTTTGGTAAGCAGAACTGGCGATGAG
GGATGCTCCTAACGTCGAGCTAAGGTGCCAAACTACCCGCGTATGAGACCCCAAGAAAGGTGTTGGCTCATTTAGACAGC
AGGACGGTGGTCATGGAAGTCGAAATCCGCTAAGGAGTGTGTAACAACTCACCTGCCGAATGAGGTAGCCCTGAAAATTG
ATGGCACTTCAGCGGGTGACTGATGCTCGGCCTGTACCACAACGGGTGTGGTATGGAGTAGGGGGGTCTTCTGGTTGCGT
GGAAACATCGCTGAGGAGCGGTGCGGAGCGGCTAGAAGTGCAGATCTTGGTGGTAGTAGCAATTAATCAAGTGCGACCCT
TGATGACTGACGTGGGGAAGGGTTCCATGAGAACATCATTTGGCCATGGGTTAGGCGGTCCTAAGGGAGTGACATTGTCA
AAAAAGGAAGGCTCTTGAGCTTTCGCCCGACAGGGCAACTGGTTAATATTCCGGTCCGACATAGTTGTCTGAGTGGCAAC
ACGACAGTACCCGAATTAGTGTTTGGTTGGCGCCGAGAAGCGTTTTCTTTGCTTTCTAACAACTGGCAACCCTGGAATCA
GCTCAGCTGGAGAATGGGTTTCGCGGTTGGAAAAGTGCCTCGTTCTTCGGGGACTTCGGACTGTCACCAAACATCCGTGA
AACTTCGGGGTCTGTAGTCGACTATGTCTCCGTACCGAATCCGCATCAGGTCTCCTGGGTTTACAGCCTCTGGTCTTTGG
CAGACAGTAGTTAAGGGAAGTCGGCAAATTGGGTCCGTAACTTCGGGAAAAGGACTGGCTCTGAGAACTGAGTGGGGGAC
CCCACATTTGGGTGTCTTGGTGTGCTGAGGGGCGTTTCAGTTAGCTGCGACGGACCAACGTGTGCCTTTTCGTCCTGTGC
GGGCTACGCCACGGGTGACAGTCAACTCAGAACTGGAGCGGACAAGGGGAATCTGACTGTTTAATCAAAACAAAGCAATG
CGATAGTTGGAGACAATGATTACGCATTGTGATTTCTGCTCAGTGCTCTGAATGTCAACGTGGTGAGATTCACGAAAGCG
CGGGTAAACAGCGGGAGTAACTATGACTCTCTTAAGGTAGCCAAATGCCTCGTCATCTAATTAGTGACGCGCATGAATGG
ATTAACGAGATTCCTACTGTCCCTAACTACTCGCCAGCGAAACCACAGCCAAGGGAACGGGCTTGGCTTTATCAGCGGGG
AAAGAAGACCCTGTTGAGCTTGACTCTAGTCCGACTCTGTGAAATGACTCAGGGGGTGCAGCATAGGTGGGAGCGTGAGC
GTCCTTGAGATACCACCACTCTTGCTGTTATTTTACTTATTCCGTCGCGTTGGTCATGCCCTAGGGCATTGGTTTGGACT
AACGAGCGTGAGCTTTGATCTTACTCGGAAGACACAGTCAGCTGGGGAGTTCGGCTGGGGCGGCACATCTGTTAAATCAT
ACCACAGGTGTCCTAAGGCAAGCTCAATGAGAACGGAAATCTCATGTGGACCAAAAGGGGAAAAGCTTGCTTGATTCAGA
TTTTCAGTGCGAATACGAACTGCGAAAGCGTGGCCTATCGATCCTTTAAACTGAGAAATTTTCAGCTAGAGGTGCCAGAA
AAGTTACCACAGGGATAACTGGCTTGTGGCAGCCAAGCGTTCATAGCGACGTTGCTTTTTGATCCTTCGATGTCGGCTCT
TCCTATCATTGAGACGCAGAAGTCTCAAAGTGTCGGATTGTTCACCCGCCAATAGGGAACGTGAGCTGGGTTTAGACCGT
CGTGAGACAGGTTAGTTTTACCCTACTGATCATTCGATATTGGAACAGTAATCCAACTCAGTACGAGAGGAACAGTTGGT
TCAGGTTTTTGGTGTGCAGCTGGTTGAGACGCCAGTGCTGCTGCGCTACACCTGTTGGAGGATGACTGAACGCCTCTAAG
TCAGAAACCGTTCTGTGACAGTGTTGCCCACCACCCCACCGGTGCAGCGTTGATGGTTAGAGAGAATTGTTCTGCTGCGG
TCTGTTAGTTCGTGCCGCTGGAAATGATATTGATTGCCGTGGGTGTGATACATCAACTGTAAACGACTTTGTTGCAACTA
GGCATTGTAAGTCAGAAAGTAGACTTTGTTCTACGATCTACTGAGATCCAACCTACGTTGCTCGATGTGAGCTGGTCCCT
GTTCGCTCGCCGCTACTAGGGGATTCCCATTTGGTTTCTCCTCCTCCGCTTGAGTTATATGCTTAAATTCAGCGGGTATA
CTGGCTCCACTCAGTTGAATTC
>ex_Echmepteryx_hageni_58S__SRR1821982
catgcttggctacataaatttacacacgaatctaatcgatttaaaactcttaacgatggatgaCTCGACTCTCGTAACGA
GGAAGGACGCAGCAAACCGCGATAAGCAGTGTGACTTGCGTATACTCGTGAACCACCAGATTTCTGAACGCATTTGTACT
CATAGCTTGTCTATGAGTATACTTGGTTCGGTATCATTTAGATCTCCACATCTATCACTTGTGATTGAAGTGGGTCTTGA
GCTCTCTGCTCCGGCAGTTAGCTGACTTTGTGTGATATTCAACTTTCTCCACTCACAGTAATTTCTCTGAAAGTGGTCAG
TCTGTTGAAAGTCATAACAGATTTCCATGATCCGAACTCAAGTAAGGCTACCCGCTGAATTTAAGCATATAATTAAGCGG
AGGAAAAGAAaCCAACTGGGATTTCCATAATAGCGGCGAGCGAAATGGAAT
>LSU_ex_Scatopsidae_Gen_sp._GD3__SRR6155939
TGAGTTGAGTAAGGTTACCCGCTGAACTTAAGCATATCATTAAGCGGGGGAAAAGAAACTAACTAGGATTCCTTCAGTAA
CGGCGAGTGAACAAGGAAAAGCTCAAATTGAAAATCGGTAGTCTATACTGAATTGTAGTCTTCAGGAGTATATCCAGCAA
AATAGTTGGTTTGTGTCCTTTGGAATAAGGCAGCAGTGAAGGTGACAATCCTGATCGGATCAACAAATTTTGCTCCCGGA
ATACTCTCTCGAGTCGCGATCCTTGTGATTGGACCGCAAAGTAGGTGGTAAATTTCATCTAAAGCTAAATACTAACGAGA
CACCGATAGCATACAAGTACCGTGAGGGAAAGATGAAAAGAACTTTGAGAAGAGAGTTAAAAGGAACTGAAACCGCTGGA
ATCGAAGTGAAAGCCTTGTAGAGCCTAGTGAAGAGCTGTCAAAGCTGTTCACTGTGGCAATGGTGGTAATTCGGTTGTGC
GCAATGGCGGTTTCGCGTTAGCGGTTCCGTAGTGCTGGACATCGGGCTATTCACCATTAGTACAACGTTTTCTTCCCGTC
TTGTAACACGGACCAAGGAGTTCAATTTGAATGCGAGTGCAAAGGTGGTAAACCTAGGCGCGTAATTAAGGTAATGTTAA
TAAGATTCGTCTGAACTAACGTCCACCTGCTAAGGTGCGATTTAGCATTCAAATTGAGACCCGAAAGATGGTGAACTATG
CCTGAGTGAAGTGAAGCCAGAGGAAACTCTGGTGGAAGCTTAAAACGATACTGACGTGCAAATCGTTCGTCAAACTTGGG

TATAGGGGCGAAAGACTAATCGAACCATCTAGTAGCTGGTTCCCTCCGAAGTTTCCCTCAGGATAGCTGAAGTTAATTAG
TTGCGTGTGGTAAAGCGAATGATTAGAGGCATCGGGGTTTTCGTAACCTCGACCTATTCTCAAACTTTGAATCTGCGCGA
TACAGAGGTTTCTTTCTTTGAACCGCTGTCTGAATAGTAACTTCTAGTGGGCCATTTTTGGTAAGCAGAACTGGCGATGA
GGGATGAACCTTACGTCAAGTTAAGGTGCTAAAATTCCACTCATAAGATACCATGAAAGGTATTAGTTCATTTAAACAGC
AGGACGGTGGTCATGGAAGTTGAAATCCGCTAAGGAGTGTGTAACAACTCACCTGCCGAATGAACTAGTCCTGAAAATGG
ATAGCGCTGAAGTGGGATACCAATACTTGACCGTTGAATTGATTTGTTGGTTCAACGAGTAGGAGGGCGTAAGGGTTATG
GCGAAGTGATGGGTGTAAACCTTCATGGAATAGCTCTTAGCGCAGATCTTGGTGGTAGTAGCGTATATTCAAATGAGAAC
TTTGAAGACTGAAGTGGAGAAGGGTTCCATGTGAACAGCAATTGGACATGGGTTAACCGGCCCTAAGGAATAAGAGCGAG
TCTTCACTAACAGAGGTTATACTCTGTGTATTCCGAAAGGGATTCAGGTTGATATTCCTGAGTTAGGGGGCGGAGTTATG
TGGTAACACAACGAAGCTTGGACACGCAAACAATGACCCTGTGAAGCGTTTTCTTTGCTCGACAACAGTGAATTTCCCTT
GAAATTAGGTCATCTAAAGCTAAGGGAAGCGAACTGGAATGCAATCTCACTTCGAGGTTGACGGTGTGTCATTGGTTGCC
CCTGAAAATCCAAGTGAATTTTATTTCGTCCCTTATCCGTACCGAAATCCGCATCAGGTCTCCTAGGTTCATAGCCTCTA
GTCAGTAGACTAAAGTGGTTAAGGGAAGTCGGCAAATTAGATCCGTAACTTTGGAAAAAGGATTGGCTCTGGGGACTGAA
TGCGATTCTTGGTTTGATGTACGCATAGTTGTGTCGAGTGCTTACGGGTAACCGTTTGAACTTGTACGCGTCTGTGCAAT
GCGCTGAATTTGAGTAATTGCATAATCAACAGTTAACCCAGAACTGAAGCGGACAAGGGGAATCCGACTGTTTAATTAAA
ACAAAGCATTGTGAAAACCGGCAATGGTTCTGACACAATGTGATTTCTGCCCAGTGCTCTGAATGTCAATGTGGTGAAAA
TCAGTCAAGCGCGGGTAAACGGCGGGAGTAACTATGACTCTCTTAAGGTAGCCAAATGCCTCGTCATCTAATTAGTGACG
CGCATGAATGGATTAACGAGATTCCTACTGTCCCTAACCACTATCTAGCGAAACCACAGCCAAGGGAACGGGCTTGGAAT
AATCAGCGGGGAAAGAAGACCCTGTTGAGCTTGACTCTAGTTTGACTCTGTGGAGTGGCTTAGGAGGTGTAGAATAAGTG
GGAGCGCAAGCGTCGGTGAAATACCACTACTCCTAGAGTTACTTTACTTAATCCATTAGGTTGTAATTCATTTTATGAAT
TCTTGATCTAAGGCAATTTTTTTGCTGATCAGTGTGGATGACACAGTCAGATGGGGAGTTTGGCTGGGGCGGTACACCTG
TTAAACGATAACACAGGTGTCCTAAGACAAGCTCAATGAGAACAGAAATCTCATGTAGACTAATAGGGGAAAAGCTTGTT
TGATTTTGATTTTCAGTACGAATATAAACTGTGAAAGCATGGCCTATCGATCCTTTAGACTTGAGAAATTTTCAGCTGGA
GGTGTCAGAAAAGTTACCACAGGGATAACTGGCTTGTGGCAGCCAAGCGCTCATAGCGACGTTGCTTTTTGATCCTTCGA
TGTCGGCTCTTCCTATCATTGCGGACGCAGAAGTCCCAAAGTGTCGGATTGTTCACCCGTTAATAGGGAACGTGAGCTGG
GwTTAGACCGTCGCGAGACAGGTTAGTTTTATCCTACTGATGATGATTTCGCTTCAGTAATCCAGCTTAGTACGAGAGGA
ACAGCTGGTTCAGGTACTTGGTTGTGTAACTAATTGAAAAATTAGTGTTACAACGCTAACATCTGTTTACGAAAGACTGA
ACGCCTCTAAGTCCGAAATAAAGCTGATAGCGAAGTTTTGTATTGCTGTGGTTAATCGATGGGATTATACCTAACACATA
CCCCGGGCTTCGGCTTGTGATAAATGTACTGTTAACCATTTGCGGCTCGTATCACGAAGACGACGTGGATGATTACAG
>Paralecudina_sp._ex_Marine_sediment_metagenome__SRR8157148
ATGAAGGGGTGAAGGGGTGGTGGTGAAGGGGTGGTGAAGGGGTGGTGAAGGGGTGGTGAAGGGGTGGTGAAGGGAACAAA
GATATTCGGGTGAAGGGGTCCTGGAGGGCCTGGAGGCTGCAGCACCTGGTTCGCAGTGAGGCTGCAGCAGTCCTGGAGGG
CCTGGAGGCTGCAGCACCTGGTTCGCAGTGAGGCTGCAGCAGTGGCTGCAGCACCTGGTTCGCAGTGAGGCTGCAGCACC
TGGTTCGCCTGTCGGCTTCGGCGCTGCAGCACCTGGTTCGCAGTGTACGTTCGGCGCTGCAGCACCACCTGGTTCGCGCT
GCAGCACCTGGTTCACCACCTGGTTCGCGCTGCAGCACCTGGTTCGCAGTGTACGTTCGCCTGTCGGCTTCGGCGCTGCA
TCACCACCTGGTTCGGCGCCGAAGCACCCCTGCTTCTGCGCCGAAGCAGCCTCCGGGTATGCTTCACCGGTTATTGAGTT
CCGGTTATGCCTTCTGGAGAGTTTACCGGTTATTGGGTTCCGGTTATGCCTTCTTTTCCGGTTATTGAGTTGCGGTTATG
CTTTCTGCCATGCAAGTAGTTGGTTGGAACCAGTTTACCGGTTATTGAGTTCCGGTTATGCTTTCTGGCACGCAACCTAC
TTGGTTCCAGTGGGGGTTCCAGTCGGTAAACCTACTGAATACGTACGGTTATGGAAGTAACTGGCGCAGAACGGATCAAA
GGAGGGTTAATTAGTACTATTTGCGTAGAGTGTTATTGAAAGGAATAGTGGAGCTGCATTGAGACGTATGTCACGGTGCG
CGCAGTCCAAGGTGTTCTGATGGGGTTGTATGGTTAGAAGGTTTCGAAATTATTGGTTAGAGTTGTGGGTGAACACCCGT
TGGTGCTTGTCGTTCGAACTTATGCTGCCTAGAGCTTGCTTTGTTCTAGGTTCTTCTTTGTAGGACTTTTGTGTCCTGGT
TGATCCTGCCAGTAGTCATATGCTTGTCTCAAAGATTAAGCCATGCATGTGGAAGCATCAGCTTCATATCCGCAAAGCTG
CGAATGGCTCATTAAATCTGCGCTAATCTGTCCAAGAGTCACACAGATGGATAATTGTGCTAATTGTGCAACTAATACGT
GCTGAATCCCAGGTTTCCTGGGGCACTTATTAGAATAAGAACCGTCCCGCGCAAGCGGATACTCTTTGGATGTAAGCCAT
AAGGTTGTATACCGTGATTTTTTCACTGGTATCTTGGGTGAGGCGCAGCCCTATCAGTTGCGGACGGTAAGGTCTTGGCT
TACCGTGGCGTTTACGGGTGACGGGGAATTTGGGTTCGATTCCGGAGAGGGAGCCTGAAAAACGGCTACCACATCTAAGG
ATGGCAGCAGGCGCGCAAATTACCCAATCTTAACAGAGGGAGGTAGTGACGAGCAATAACAGCGTGTGACTTATGTTGCA
CGACTGGAATGAGACGGGCTTAAAACACTCGTTGAGAATCAATTGGAGGGCAAGTCTGGTGCCAGCAGCCGCGGTAATTC
CAGCTCCAATAGCATATATTAAAATTGTTGCAGTTAAAAAGCTCGTAGTCGAACTTTGTTCAGCTGGTTGCACTTACAGC
TTTGCTGTTCATGCGACCGACTGGACTTTGCGTGGCACTCTTGTCTTTGACATTGGTGCTCCGCCACTTTGAATATATCA
AAGTGTTTAAAGCAAGTCTACTTGATTGCTCAAGCATGGAATACAGAATCGGCGTTTGCCTTTTCTAAGGTTCTAAAGGC
ATTTGCAATGATTGAGAGGAACGGTTGGGGGGATACGCATTTGACTGTTAGAGGTGAAATTCTTGGATCAGTCAAAGGCG
AACTGATGCGAAAGCGTTTCTCAAAAACGTTTTTATTGATCAAGAACGAAGGTTAAGGGATCGAAGACGATCAGATACCG

TCGTAGTCTTAACGGTAAACTATGCCATCCAAAGATCGGGCAAATGCATAGAGATTGCTCGGGATTTCGTGAGAAATCAG
AGATATTGGGTTCTGGGGGAAGTATGTGCGCAAGCCGGAAACTTAAAGGAATTGACGGAAGGGCACCACAAGGAGTGGAG
TATGCGGCTTAATTTGACTCAACACGGGGAACCTTACCAGGGCCAGACATAGGAAGGATTGACAGGATGCTAGTTCTTTC
TTGATTCTATGGGTGGTGGTGCATGGCCGTTTCTAGTTGGTGGAGTGATTTGTCTGGTTGATTCCGTTAACGAACGAGAC
CTTTTCTTCCAAATTGCTATGCATTATCTTGATAGTGTGTAGTAACTCGGAGTACAACATGTGGAAAGCATGGGGAAGAG
AAAGGCGAAAACAGGTCTGTGATGCCCTTAGATGTTCTGGGCCGCACGCGTATTACAATGGCGTTCTCACTTAGCATTAC
ATCCCTTGTAAAAGAGGGAGGTATCTTATAAAATAACGCCAAAATCGGGATTGGCTATTGGAATTATTGGCCTTGAACGA
GGAATTCCTTGTAATTGCAAATCATCAGTTTGTGATGAATACGTCCCTGCCCTTTGTACACACCGCCCGTCGCTCCTACC
GATTGGGTGATCCGGTTAATTCCATGGAGTGCCTTTGTATCCTCACGGGTGCATTTGGTGCAAAATGGTGTAAACCTTAT
CATTTAGAGGAAGGAGAAGTCGTAACAAGGTTTCCGTAGGTGAACCTGCGGAAGGATCATTCTTTTTTTGGGCAGATGAA
TGCACGGTTATCCGATATCGGTCATTGATTTCCGGATAAAGGTGGGCTACGATCATGACAGGACTTCCTCACATTGAGGA
GTGTTCAGTATATGCATAGATTAGTTCAAATTGGCAGCTGCTACTAGCAGCTGTGCATGGCAAGCGTAAATTTCAAATTG
TTGGAACCAGCTGCTCTGAAAAGCGTCAATTTTCAGCTGGCACCAACTCTTTTCGGCTAGTGTATCGGCAAGCGTTCAAA
TGAAATCAGTGAGAAGGGTTCTGACGATATCTGAGAAGTGTTCTGACGAGATCTCAACGAAGCGTTCTGACGGGATCTCT
TTGTGAAGCGTTCGGACAAACTTTCTGGCAAGTGTTAGCCTTTCTTTTGTCGACTAGTGTTTACAATAGCATACGGTACC
TATAATATCATATTCATAACTTGCGCAACGAGTCGCACGGACCCACAACGATGAAAATCGCAGCAGAATGCGTGACGCAT
AGTGAATCGCATTTTTCGGCGGATCTAGTCGTTTATTGAACGCATATCGGCGGAGTGCCATTTGCGGCGCTTCGCTTTTT
CCATTGAGCGCCTCATTTCAATTCTCCACCAAATTCGTTTGGTGGACCTCTGAGGAGGTTGTTTCTTTCCCATTATCTCG
GTAGTGGTGAAGAGAGGTGGTCTTCTTGGAACGAGTTTCCAAGTTGTCCTATTGCGGTGGTCTTCTATGATATGCAGCAC
TTGCTTCATCTTAGAATATACACGAAGTTATTCTGTCGTGGCCTCAATTGAGAAAGAAAACCCGCTGAATTTAAGCATAT
TAATAAGCGGAGGAAAAGAAACCAATAGGGATTCCCTTAGTAACTGCGAGTGAATAGGGAAGAGCGCATTATGCTAATCA
ACATTCTTTGTTGAATTGTGGTGTATACTGGCAGTTGCAGATGGAAGTGCTTATTCTTCTGGTTTTTTGGAAAAAGAACC
TATGAAGGGTGAATGGCCTTTTTGAAGGTAAGTGCTTGCATCTTTGTAGTTGCCTTTTGCCGAGTCGTACTCTTTGAAAG
TAGAGTGCAAAGATGGGTGTTACGTTTCATCCAACGCTAAATATATGTGCGGTTCCGTTAGTCAACAAGTACCGTGAGGG
AAAGTTGAAAAGAACTTTGAAGAGAGAGTTAAAAAACGTGACCTTGCGCAAGTGAAGTCGAACTTAGGGCTGTATCAGTG
GATCACCTTTCAATGCATTCTATGTTGTTGAAGGTGTTCTTCCGCCCCCCGGAGTACTAAGGAAATTGAGCGAACCTCCA
AATTCGAACTTGTTTGAATCCCTGAAGGCGAGTGCCACATATCTTTATAGTATTTCCGGAGCAAACCTAAGTGCGTCCCG
TCTTGAAACACGGACCAAGGAGTTTAGCTGTGATGCGAGTAGCCTATTTCAGTAGATATGGTTGCGATTCGACGGAGAGC
TTTCACTTTTGCAAAAAAGTGATCGGACGTGATTTGCGCTAGCATTTCATGTTACCGGAGCATCACTGCTAAGACCCGAA
AGATGGTGAACTATTCTTGAGTGAGGCGAAGTCAGAGGAAACTCTGATGGAGGCTTGTTACGTTACTGACGTGCAAATCG
TTCGTTGAACTGATGAATAGGGGCGAAAGACCAATCGAACCATCTAGTAGCTGGTTCCCTCCGAAGTTTCCCTTAGGATA
GCTGGAGGTGTGACAGTTCCGTGCTGTAAAGCTAATGATAAGAAGTTGCAGGGGATTAGTATTCCTTGCCTTATTGTCAA
ACTTTTAATGTGCGGTATGTTTTGCTTCGTTTAGAAGCATCACGTAGAATGTTTGCTTCCATGTGGGCTTTTTCTGGTAA
GCAGAAATGGCGATGAGGGATGCACCTAAAATTATGGTAATCGGACAAAAAGCCATTTGCTGAGTAACCATGAAAGGTGG
TGGTCGATTTTGACAGCAGGACGGTGGCCATGGAAGTCGGAATCCGCTAAGGAGTGTGTAACAACTCACCTGCCGAATCG
AGCTCCCCTGAAAATGGATGCCGTTAAAATGTCTATCGTGATCCATGATTCGCAGTAAAGCGTTCAACTGCGAGGTAGGA
GGGCGCGATGGTTGCGGTGAAGTGTTGTCGTGAGGCGGCATGGAGCGACAATCGGTGCAGATCTTGGTGGCAGTAGCGTT
TACTCAAAAATGAATGTCGAGGACTGATGTCGGAATAGGTTTCCATGTTGACAATATTTGAACATGGGTTAGGCGGTTTT
AAGCGATTTCCTCGCAAGAGTTTTCGCGAAAAAGCAACAGGTTAATATTCCTGTGCTGACAACATGCGGCGCTTTTGGTA
ACAAATGTTCTCCAGAACTAATAAGCATCTCCCCCGAACAGCGATACCTTTGTTGTGAAACGGTTGTTCACCCTGGAAAT
GTATTGTACAACGAAGGGGTTGAAGCACCGGCATACCACCTGAATTCGTTTGGGTGCGGAGTGGTTGTGCTTGTCGYAGA
AAGTTTTGGAGTTCAATTGAGTGTGGTATGTTTTTAGCCGTACCTTAAACCGCATCAGGTCTCCGAGGTTCATAGCCTCT
AGTTTTAAGAAGAAAGTAGGCAAGGGAAGTCGGCAATTTCGATCTGTAACCTTGGGAGAAGGATTGGCTCTGAGAATTGG
CTGCTTATTGCTTCCTTGTGGGTAGCAAAGGTGATTGTTCTTCCGTCTGCTCGAAAGGGCTCTCGAAAAAGCATTCGCAT
TGCATTCCTGCAAGCCCGTAAGGGTTTAGAAGTATAGCAACCGCTTTATTTCTCAGAACCGGAACAGACCAGGGGAATCT
GACTGTTTAATTAAAACAAAGCATTGCGATAATCCCTCATTGGGGTCTTTACGCAATGTGATTTCTGCCCAGTGCTCTGA
ATGTCAATGTGAAGAGATTCAACCAAGCGCGGGTAAACGGCGGGAGTAACTATGACTCTCTTAAGGTAGCCAAATGCCTC
GTCATCTAATTAGTGACGCGCATGAATGGATCAACGAGATTCCTACTGTCCTTGTCTACTATCTTGTGAAACCACAGCCA
AGGGAACGGGCTTGGTATAAATCGGCGGGGAAAGAAGACCCTGTTGAGCTTGACTCTAGTTTGGTTTTGTAAAGTGACTT
AAGAGGTGTAGTTATAAGTGGGAGCTCCGGCAACAATGAAATACCACTGCTTTTAATGTCATTTTACTTATTCCTTTTCG
GGCATTCCGGATACCCGGTTCATGTCCAAAGCCGCTTGCGGTGATGCGATAGGAAGACAGAACCAAATGGGGAGTTTGGC
TGGGGCGGCACATCTGTTATACAATACCACAGGTGTCCTGAGGCAAACTCAGTGAGAACAGAAATCTCACGTAGACCAAA
AGGGGAAATGTTTGCCTTATTTGCACTTGCAGCAAGAGTGCAAATGGTGAAAGCCTGGCCTTTCGATCCTTTATGCTCTG
GAAATGTTCCAGCTAGAGGTGCCGGAAAAGTTACCACAGGGATAACTGGCTTGTGGCAGCCAAGCGTTCATAGCGACGTT

GCTTTTTGATCCTTCGATGTCGGCTCTTCCTATCATTCTGACGCAGAAGTCAGAAAGTGTGGGATTGTTCACCCATTAAT
AGGGAACGTGAGCTGGGTTTAGACCGTCGTGAGACAGGTTAGTTTTATCCTGCCGATTATTAGCGTCAATGCAGCAATCC
AACTCAGTACGAGAGGAACAGTTGGGTCAGCAAGCTGGTGTGCAATGAGTAGTTTCGCTGACGTTGCATGACTTATGCTG
GTTGCGGATGGCTGAATGCCTCTAAGCCAGAAGCGATGCTGTATTATTTATGTGGCGCTTATTTTCTAGCAAGATTGTAT
GGTTGCCATAGATCTATTGCTTCCATTCCTATCAATAGTCAATTGATATCAATGGAGATAAGTTCTGTTATATTTGCTGG
AGGTATTCGAGTAGATGACTTGGATTTTTTTGGCATTATTCGCTGAGAAGCATTCTTTATTGCGACTCGCTGAGGTGCCT
TGCAGCATGAGTCTCCCGGTTATTGGTTTCCGGTTATACCTGGGC
>Lecudina_tuzetae_SSU_assembled_SRR8980210
ATCAACGCAGAGTACATGGGAACCTGGTTGATCCTGCCAGTAGTCATATGCTTGTCTTGCAGATTAAGCCATGCATGTCT
TAGTGTAAATATTTTACAGTTAAACTGCGGATGGCTCATTACAACAGTTATCGTCTGCACGGGCAATCTTTCTACGGATA
TCCGTGGAAATACTACAGTTAATACGTAACACAAAATTTCGGTTTTGTGTCACATATTACTGTGAGGAGATGGCAAGGTT
TACCTTGCCCTCCAGGAATACTTCTATGTAAGAATCGCACTTCATGTGCGAATGCCCGTCAGAGTTTCTGACCCATCAGG
TTTCGACGGTAGGGTATTGGCCTACCGTGCCTATAACGGGTGACGGGGGATTAGGGTTCGATCCCGGAGAGGGAGCTTGA
GAGATGGCTACCACATCTAAGGAAGGCAGCAGGCGCGCAAATTACCTATCCTTGACTCAAGGAGGTAGTAAATAGCAATA
ATGACGCATGATTTTAATCGTGCGATCGGAATGAGTGGTTTCTAAACCAAACCACGAACTCAATTGGAGGAAAAGTCTGG
TGCCAGCACCCGCGGTAATACCAGCTCCAATAGCGTATATTATTGATGTTGCAGTTGAAACGTTCGTAGTCGAACTTTTT
CTGTTAAAGACATGTGTCTGTCATAGACAGCATATGTTCTTATTGCAGAAAAGTGTTAATTCGTCTATGTGTTCCATGTA
GGCGTCTATTAACACTGTACTTTGAGTGAATCGGCGTGTTCCACGCAGGCTTTACGCCTTGAACGATTTACCATAGTATA
TCCATAGTATAGAGATGTCATCTTGTCTTCTCGATGGCATTTTTGATACTTAAAAGAACGGTCGAGGGCATTCGTATTCA
ACTGCCAGAGGTGAAATTCTTTGATTAGTTGAGGACGAACCATTGCGAAAGCATCTGCCAAGGACGTTTCTGTTGATCAA
GAACGAAAGTTAGGTGATCGAAGATGATTAGATACCGTTGTAGTCCTAACTATAAACTCTACTATCCAAGAATTGGATTG
GTGGCTTTACGCCTCTCCAGTACTTTCTGAGAAATCAAAGTCTTTGAGTTCTGGGGGGAGTATAGTTGCAAGGCCGAAAC
TTAAAGGAATTGACGGAAGGGCACCACCAGGAGTGGAACTTGCGGCTTAATTTGACTCAACACGGAAAAACTTACGAGGA
TAAGACAAACGAAGGATTGACAGATTGATCGCTCTTTCTTGATTGTTTGGGTGGTGGTGCATGGCCGTTCTTAGTTCGTG
GATTAATTTGTCTGGTTAATTCCGTTAACGAACGAGACTTTGACTTACTAGTTAGATTGCTTTATTCATAAAGGAATCTT
CTAGAAGGACCATGTGTTCCTAACACAAGGAAGCGCGAAGCAATAACAGGTCTGTGATGCCCTTAGATATCCTCGGCAGC
ACGCGTGTTACAATGATGTATAGAACATGTTTCGCCCTTCGAAAGAACGGTGTAATCATTAATATGCATCAACCCAGGGA
TTGTTTGTTGTAATGTCAAGCATCAACGAGGAATTCCTAGTAAGCGTGCATCATTAATCCGCGTTGATTACGTCCCTGCC
CTTTGTACACACCGCCTGTCGCATCTATTGATTAGGTTGTATCATGAGTTGAGCGGAGATGATTAAAGGATTTTTCTTTT
ATTTATCAAAGCTTTGCAAATGGTATTACCCAGAAACAGATAAAGTCATAACACGGCAGCTGTAGGTGAACCTGCAGCTG
GATCATTAATAGTTGACAATATAC
>Lunidium_terebellae_SSU__SRR21755681
GGTATCAACGCAGAGTACATGGGAACCTGGTTGATCCTGCCAGTGAACCTGGTTGATCCTGCCAGTAGTCATATGCTTGT
CTCAAAGATTAAGCCATGCATGTCTAAGTATAAGCTTTTATACAGCAAAACTGCGAATGGCTCATTAAAACAGTTATAGT
TTATTTGATGATCAAATTACATGGATAACCGTGGTAATTCTAGAGCTAATACATGCGAAAACACCCGACTTTTTGGAAGG
GTGGTATTTATTAGATAAAGAACCATTAGTCTCTTTACGGGGGCTTTTCTGGTGATTCATAATAACTTAACGAATCGTTT
TCTTAAACGATAGATCATTCAAGTTTCTGACCTATCAGCTTCGACGGTAGGGTATTGGCCTACCGTGGCATTGACGGGTA
ACGGGGAATTAGGGTTCGATTCCGGAGAGGGAGCCTGAGAAACGGCTACCACATCTAAGGATGGCAGCAGGCGCGCAAAT
TACCCAATCCTGACACAGGGAGGTAGTGACAAGAAATAACAACGCAGGGCATAAATTGTCTTGTGATTGGAATGAGTAGA
ATCTAAAACCCTTTACAAACTCCATTGGAGGGCAAGTCTGGTGCCAGCAGCCGCGGTAATTCCAGCTCCAATAGCGTATA
TTAAAGTTGTTGCAGTTAAAACGCTCGTAGTTGAACTTCTGCACAGATTTACGGCTTCGACTTCTATTTAGTTGTTGTTA
AGTTTGTATATTTGTGTATCTTCATGTTGTTTGGTATATTTTGGTTGTTTTATTACATCCATGTATACATTATAAATAAT
GACATGACATTTACTTTGAGAAAATTAGAGTGTTTCAAGCAGGTTTTTTCTGCCTTGAATACTTCAGCATGGAATAATAA
GATAAGACTTTGGTTTTATGTTATTGGTTCAAAAACCTTAGTAATGATTAATAGGGACAGTTGGGGGCATTTGTACTTAC
CTGTCAGAGGTGAAATTCTATGATTAGGGAAGGACAAACAACTGCGAAAGCATTTGCCAAAGATGTTCTCATTAATCAAG
AACGAAAGTCAGGGTAATGAAGACGATCAGATACCGTCGTAGTCTTGACCATAAACTATGCCGACTAGAGATTGGAGGTC
GTCATTTCTTATCGACTCCTTCAGCACCTTATGAGAAATCAAAGTCTTTGGGTTCCGGGGGGAGTATGGTCGCAAGGCTG
AAACTTAAAGGAATTGACGGAAGGGCACCACCAGGAGTGGAGCCTGCGGCTTAATTTGACTCAACACGGGAAAACTCACC
AGGTCCAGACATGGGAAGGATTGACAGATTGATAGCTCTTTCTTGATTCTATGGGTGGTGGTGCATGGCCGTTCTTAGTT
GGTGGAGTGATTTGTCTGGTTAATTCCGTTAACGAACGAGACCTTAACCTGCTAAATAGTGACACGAAACTCGTATTGCT
ATATGTTATATTCACTTTAACCGGTGTGTTTTCATATATGTGGTTGACTGGAAGCTCATATCTTGGTATCGTCTTCGGGT
GTTGTCTTGGTATGTGCTCTAGTTGTTTGTGTATCACTTCTTAGAGGGACCGTCGCGTGTCTAACGCGTGGAAGTTTGAG
GCAATAACAGGTCTGTGATGCCCTTAGATGTTCTGGGCTGCACGCGCGCTACACTGATGCATCCAACGAGTTTTTTTTCT
CCTTATCCGATAGGATTGGGTAATCTTGTGAGTATGCATCGTGATGGGGATAGATTATTGCAATTATTAATCTTCAACGA

GGAATTCCTAGTAAGCGCAAGTCATCAGCTTGCGCTGATTACGTCCCTGCCCTTTGTACACACCGCCCGTCGCTCCTACC
GATTGGGTGATTAGGTGAATTATTCGGACCACACGACGTGTGTAATTCTTGTGATTACCGTGTCATCGTGGGAAGTCTTG
TGAACCTCATCGTCTAGAGGAAGGAGAAGTCGTAACAAGGTTTCTGTAGGTGAACCTGCGGAA
>Lunidium_terebellae_LSU_SRR21755681
GTATCAAGGCAGAGTACATGGGCAAGACCTGAATTCGTGCAAGGCTACCCGCTGAATTTAAGCATATAATTAAGCGGAGG
AAAAGAAACTAACAAGAATTTTCTTAGTAATGGCGAATGAACAGAAAACAGCTCATACTGAAAATTGGCCTTGGTTTATT
TTTAAACCGAAGCCGAATTGTAGTCTGTAGAAGTATTTCCAGTAAAACCTCAGAGTAAAGTCTCTTGGAAAAGAGCGCCA
AAGAGGGTGACAGTCCCGTATTTGCACTCTGGGTCATAGTCGTGTTTTACGTACGGAATGCTTTCAACGAGTCGTGTTCC
TTGGGATTGGAGCACAAAATAGGTGGCTTATTTCATCTAAAGCTAAATATCGGCGGGAGACCGATAGTAGACAAGTACCG
TGAGGGGAAGATGAAAAGGACTTTGAAAAGAGAGTTAAAAGAATCTGAAATTGctGAAAGGGAACCGAATGGAAGTTGTT
AACTGATGAGATTTCGTGGGTTTCAATGTTTTTATTAATATTGATTTACACGCTTGCTTATCGGTTGAATCAGATCATAT
TATTATTTCATATATGACGTCGTTGCTCTTGCTTTTGCATACCGAGATTTCGGTTTCGGTTGTGCCAGTATATGATGCCA
ACACCGTCTAGGATGATATGACTTCTGATTTCGTGCATTCGTCAACAGCTTTCATCCGACCCGTCTTGKAACACGGACCA
AGGAGTCTAACAAATGTGCGAGTGTATAGGTGATTCAAACCTGTACGCAAAATGAAAGTGACTGGTAACAAGAAGCATGT
TTTATATATGTTCTCTGCCTTACCAACCGATCTTTGAATTGAGGTTTGAGTGATAGCGCATCTGTTAGGACCCGAAAGAT
GGTGAACTATGCCTGAATAGAGTAAAGCCAGGGGAAACCCTGGTGGAAGCTCGTAGCGATACTGACGTGCAAATCGTTCG
TCAAATTTGGGTATAGGGGCGAAAGACTCATCGAACCATCTAGTAGCTGGTTCCCTCCGAAGTTTCCCTCAGGATAGCAG
GAGTTGATTTGTTTATTTTTCTAATAGTTGTATCAGGTAAAGCGAATGATTAGAGGCCTCGGGGGTGCGTTCATCCTCGA
CCTATTCTCAAACTTTAAATGGGTACGATCCAAAGATTGCTTTGGCTTTACGTTTTGTTCGTCTTTCAAGATGTTCAGAT
TGTATGTGCACAGTGAATCTTTGGGATTAATATAAGACTCCTAGTGGGCCATTTTTGGTAAGCAGAACTGGCGATGAGGG
ATGCTCCTAACGTTTGGTTAAGGTGCCAAAATACTCGCTTATCAGATACCACAAAAGGTGTTGGTTCATTTTAACAGCAG
GACGGTGGTCATGGAAGTTGAAAACCGCTAAGGAGTGTGTAACAACTCACCTGCCGAATGAATTAGCCCTGAAAATTGAT
GGCGCTGCAGCGAGTTACCGATACCAAACCATTGTTGTGAGTAAGATACAATACCTGTGATTACATGCTATATTTGTTGT
ATAACAATACTTTAGCAAAGCTTTACAGCTATAAATAATTTTTACAACAATGAGTAGGAGGGGGTGGCGGTTGTATAGAA
GTAATCAAACGTGAGTTTGTATGGAACAGCCGTTAGTCCAGATCTTGGTGGTAGTAGCAATTATTCAAATGACAACTTTG
AAGACCGAAGTGGGGAAGGGTTCCATGTGAACAGCAATTGGACATGGGTTAGCCGGTCCTAAGGGATAGGAAAGCTCTGA
TACTTTATTCTTTGCAAGGCTTTCTTTTGATTGCCTGCGATTCCCGAAAGGGAATTAAGTTAATATTCTTAAGCCAGGAC
GTGGATAATAATGATTATGACATATATGGTAACATAAGTGAACTCTGATACGCAAGTGGGTGCCCCGGAAAGCGTTGTCT
TTGCTTTTTAATAGTCTCTTAAATACCCTGGAATCAGTTTATCTGGAGATAGGGTATTTGAGCTGCAATTCAGCATTTCA
TCATTTGAAATGTCCGGTGCGTTCCCGATTGCCCTTGAAAATCAGGGGGAAGAATGACGAGGTTTAGTCTTTAGATCAAG
CTTTGTTGTTATTTGCACCTCTGGCCGTACCAATAACCGCATCAGGTCTCCAAGGTTAGTAGCCTCTGGTCCATTAGACC
AAAGTAGATAAGGGAAGTCGGCAAATTAGATCCGTAACTTCGGGAAAAGGATTGGCTCTGAGGGCTGGGTGTTGGGGTCT
TTTAGTGAAGATGTTTGCTATGCTGATGAACTACGCGTCGAGAACCGCGAGGCTTTTGACAAGTGGATCATTGCAAGGCT
TCTTCGGAACTTTAAGATGAGTATGTGTATGTATATAGTACTCCTATGTATATATGTGTATCTCCTTGCCCTTCACATTA
AACAGTCATCTCAGAACTGGAGCGGACAAGGGGAATCCGACAGTTTAATTAAAACCAAGCATTGCGATGGCCATAAATCG
GTGTTGACGCAATGTGATTTCTGCCCAGTGCTCTGAATGTCAATGTGATGAAATTCAATCAAGCGCGGGTAAACGGCGGG
AGTAACTATGACTCTCTTAAGGTAGCCAAATGCCTCGTCATCTAATTAGTGACGCGCATGAATGGATTAACGAGATTCCC
ACTGTCCCTATCTACTATCTAGCGAAACCACAGCCAAGGGAACGGGCTTGGAATAATCAGCGGGGAAAGAAGACCCTGTT
GAGCTTGACTCTAGTCCGACTTTGTGAAATGACTTAGGAGGTGTAGAATAGGTGGGAGTGTTTCGGCACGACGGTGAAAT
ACCACTACTCTTACTGTCATTTTACTTATTCCGTTGAATTGGGACGCGTAATTGTTATCACTGTGCTGTTTCTATCATGG
AGCTTCCAGTGCTCTGCCCTTTGCGTGTTATTGATTTAACGTTGGTACAAAATAATCATCATGCTTTGTATGCTTTTCGG
GGTATGCTTAGTATGGTGGCCGGCAGACTTAGGCGGAAGACATAGTCAGGTGGGGAGTTTGGCTGGGGCGGCTCATCTGC
TAAACAATAACGCAGGTGTCCTAAGGCAAACTCAATGAGAACAGAAATCTCATGTTGACCAAAAGGGGAAAAGTTTGCTT
GATTTTGATTTTCAGTATGAATACAAACTGCGAAAGCATGGCCTATCGATCCTTTAGGTTTTATCTGTGAAATTTACAGC
TAGAGGTGTCAGAAAAGTTACCACAGGGATAACTGGCTTGTGGCAGCCAAGCGTTCATAGCGACGTTGCTTTTTGATCCT
TCGATGTCGGCTCTTCCTATCATTGAGACGCAGAAGTCTCAAAGTGTCGGATTGTTCACCCGCCAATAGGGAACGTGAGC
TGGGTTTAGACCGTCGTGAGACAGGTTAGTTTTACCCTACTGATGATTTTGTTATAGGTAAATAGTTAGACCTTCTTTAA
AATCTTTGATACTGCGACAGTAATCCAACTTAGTACGAGAGGAACAGTTGGGTCAGATACTTGGTTAATGCGGCTAGTTG
AAAAGCTAGTGCCGCGAAGCTACCATCTGTTGGATAATGACTGAACGCCTCTAAGTCAGAATCCGTACTGGATTGTCAAA
TGCAGTGTTATCCACCATTTCCGATAAGACATGTAAATTAATAGGCAAGTTCGCTCAAGGGCGTGTCTTTTTTTGCCGTA
TTTCACTCGACACTTTATTTGGTTTCGTCTGGTTCCAAATAGAGTCGTATGACAATTTCAATTGTTGGGAATGACACATG
CTATGTATACGACTTAGTTGTATCCGGGTATTGTACGTACGAGAGTAGGCTTTGTCTTACGATCTACTGAGATTCAGCCC
GAGTTACAATAATGTGTGATGAC
