# Supplementary material for: Cellular organization and phylogeny of Cryptonaiada gen. n. (Cryptonaiadidae fam. n.) shed light on the evolution of intracellular vertebrate parasitism in Apicomplexa
Source: mBio. 2026 Oct 5;17(11):e01957-26. doi: 10.1128/mbio.01957-26 (PMC13753809; doi:10.1128/mbio.01957-26)
Supplement: Supplementary file 2 [file mbio.01957-26-s0004.pdf]

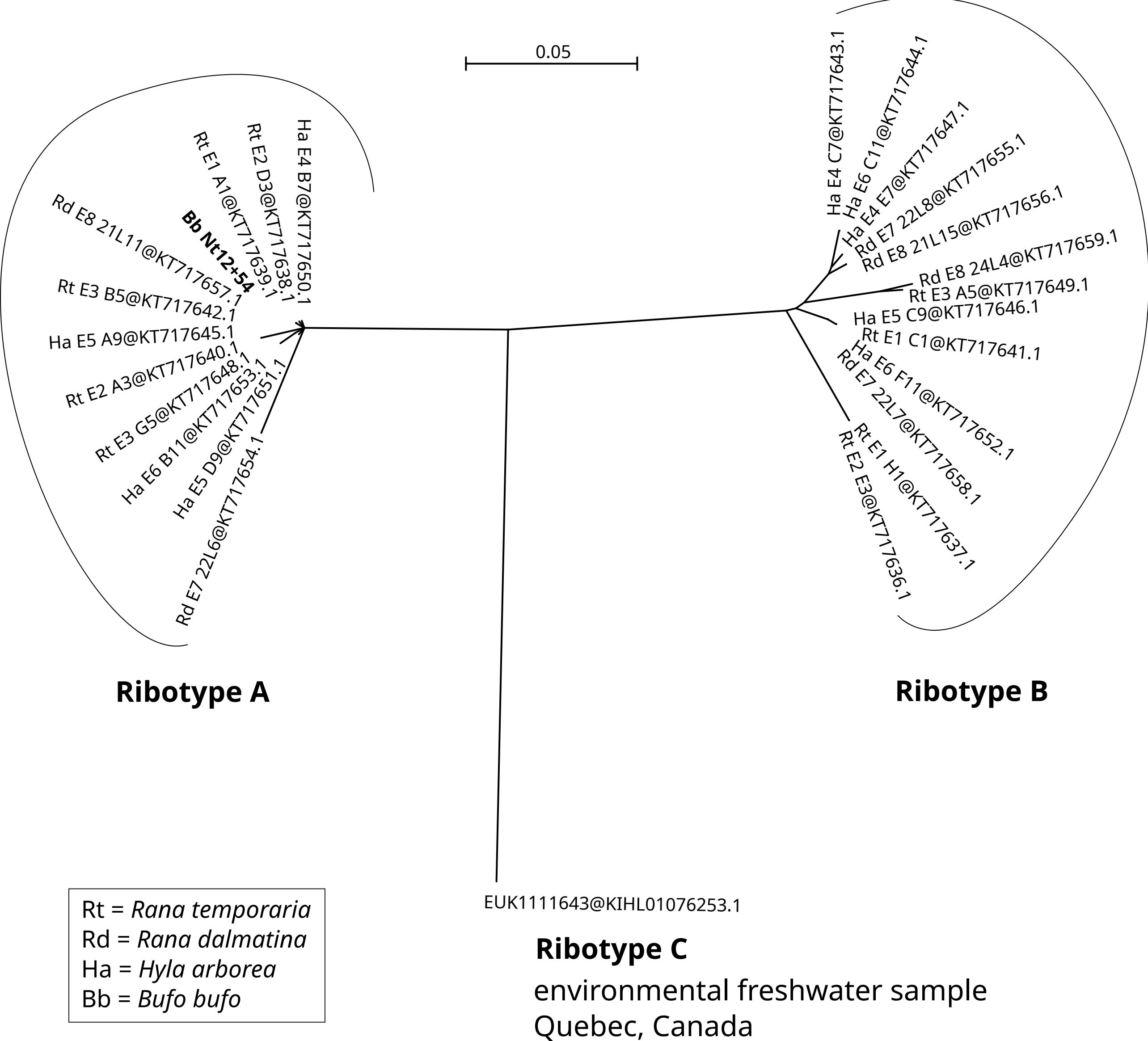
0.05
Ha E4 B7@KT717650.1
Rt E2 D3@KT717638.1
Rt E1 A1@KT717639.1
Bb Nt12+54
Rd E8 21L11@KT717657.1
Rt E3 B5@KT717642.1
Ha E5 A9@KT717645.1
Rt E2 A3@KT717640.1
Rt E3 G5@KT717648.1
Ha E6 B11@KT717653.1
Ha E5 D9@KT717651.1
Rd E7 22L6@KT717654.1
Ribotype A
Ha E4 C7@KT717643.1
Ha E6 C11@KT717644.1
Ha E4 E7@KT717647.1
Rd E7 22L8@KT717655.1
Rd E8 21L15@KT717656.1
Rd E8 24L4@KT717659.1
Rt E3 A5@KT717649.1
Ha E5 C9@KT717646.1
Rt E1 C1@KT717641.1
Ha E6 F11@KT717652.1
Rd E7 22L7@KT717658.1
Rt E1 H1@KT717637.1
Rt E2 E3@KT717636.1
Ribotype B
EUK1111643@KIHL01076253.1
Ribotype C
environmental freshwater sample
Quebec, Canada
Rt = Rana temporaria
Rd = Rana dalmatina
Ha = Hyla arborea
Bb = Bufo bufo
