# Supplementary material for: Cellular organization and phylogeny of Cryptonaiada gen. n. (Cryptonaiadidae fam. n.) shed light on the evolution of intracellular vertebrate parasitism in Apicomplexa
Source: mBio. 2026 Oct 5;17(11):e01957-26. doi: 10.1128/mbio.01957-26 (PMC13753809; doi:10.1128/mbio.01957-26)
Supplement: Supplementary file 3 [file mbio.01957-26-s0005.pdf]

Figure S1. Gregarine-like cells in tadpole liver. A–C. General view of liver sections showing the distribution of *Cryptonaiada temporaria* oocysts and gregarine-like cells. **D–F.** Single gregarine-like cell engulfed by a host macrophage. **G.** Pair of gregarine-like cells within a macrophage. **H-I.** Gregarine syzygy-like cells enclosed within a macrophage. **J.** Cells resembling early gregarine gametocysts with a visible septum. **K.** Structures resembling maturing gregarine gametocysts. **L.** Putative early stage of a pair of *C. temporaria* oocysts in cross section. **M.** Putative sporoblast. Specimen: *Bufo bufo* (A-M). LM – histological sections stained with haematoxylin-eosin (A, B, F, G, K, L) or Masson's trichrome (C-E, H-J); semi-thin section stained with toluidine blue (M). Abbreviations: *black arrow* – oocyst of *Cryptonaiada temporaria*, *black arrowhead* – macrophage, *cv* – central vein, *si* – liver sinusoid with blood cells, *white arrow* – gregarine-like cell.

Figure S2. Host-parasite interactions in *Cryptonaiada temporaria* oocysts. A–C. Different sections through the sporozoite apex and associated multimembrane tubular structure. Specimen: *Rana temporaria* (A-C). TEM (A-C). Abbreviations: *black arrow* – oocyst wall, *black asterisk* – apical end of the sporozoite, *r* – rhoptries, *t* – multimembrane tubular structure, *white arrow* – attachment of the tubular structure to the sporozoite apex, *x* – sub-Stieda-like body.

Figure S3. Relationships among internal transcribed spacer (ITS) 1 sequences of *Cryptonaiada temporaria* (formerly *Nematopsis temporariae*). Unrooted maximum likehood phylogeny of the partial ITS1 transcript newly retrieved from *Bufo bufo* tadpoles is shown among sequences generated by Chambouvet et al. (1) and an environmental freshwater sequence from Quebec.

REFERENCES

1. Chambouvet A, Valigurová A, Pinheiro LM, Richards TA, Jirků M. 2016. *Nematopsis temporariae* (Gregarinasina, Apicomplexa, Alveolata) is an intracellular infectious agent of tadpole livers. Environ Microbiol Rep 8:675-679.
