# Supplementary material for: Cellular organization and phylogeny of Cryptonaiada gen. n. (Cryptonaiadidae fam. n.) shed light on the evolution of intracellular vertebrate parasitism in Apicomplexa
Source: mBio. 2026 Oct 5;17(11):e01957-26. doi: 10.1128/mbio.01957-26 (PMC13753809; doi:10.1128/mbio.01957-26)
Supplement: Supplementary file 4 [file mbio.01957-26-s0006.pdf]

Table S1. List of sequences selected for ribosomal phylogeny analysis in Figure 6 (newly identified and *de novo* assembled sequences are shown in blue and green, respectively).

Source	Accession Numbers or SRA runs		
	SSU rRNA	5.8S rRNA	LSU rRNA
outgroup			
Toxoplasma gondii	X75453.1	X75430.1	AF076901.1
Cryptosporidium muris	AAZY02000009.1		
Porosporidae			
Nematopsis sp. ex *Nerita peloronta* & *N. versicolor*	PV470980.1		-
Nematopsis sp. ex *Pecten maximus*	PV470981.1		-
Nematopsis sp. ex *Aequipecten tehuelchus*	PV470982.1		-
Porospora cf. *gigantea* ex *Homarus gammarus*	OM811446.1		
ex *Argopecten irradians* (Atlantic Bay scallop)	OM575024	-	OM690687.1
ex *Cerastoderma edule* (common cockle)	**SRR14870539, SRR14870540, SRR14870542**		
ex *Crassostrea virginica* (eastern oyster)	**GGQE01035772.1**	**-**	**GGQE01090476.1, GGQE01102205.1**
ex *Litopenaeus vannamei* (Pacific white shrimp)	**SRR6466326**	-	**SRR6466326**
ex *Mytilus californianus* (California mussel)	**SRR5275489**		
ex *Perna perna* (brown mussel)	**GJKQ01000789.1**	**GJKQ01000789.1**	**GJKQ01000789.1, GJKQ01000202.1**
ex *Rugalucina angela* (bivalve)	OL583988.1	-	-
other Cephaloidophoroidea			
Cephaloidophora_bipinnatae	MT112171.1		
Cephaloidophora communis	HQ891113.1		
Ganymedes themistos	PQ738226.1		
Heliospora caprellae	HQ876007.1	**SRX5759508, SRX5759509**	
Heliospora cf. *longissima*	HQ891115.1		
Thiriotia sp.	PQ738233.1		
Lecudinoidea			
Lankesteria abbotti	DQ093796_1	-	**SRR1300212**
Lecudina tuzetae	**SRX5759501, SRX5759502**		
Pterospora schizosoma	DQ093793.1	**SRX5759500, SRX5759511**	
Ancoroidea			
Ancora sagittata	KX982503.1		
Polyplicarium lacrimae	JX535344_1	**SRR21755685**	
Polyrhabdina pygospionis	MT214481.1		
Trollidium akkeshiense	MN381955.1		
Paralecudinoidea			
Paralecudina sp. oshoro	OQ650278.1		-
Trichotokara japonica	JX426617.1	-	-
Environmental sequence DSGM-8	AB275008.1	-	-
Environmental sequence PS13B11	GU072504.1	-	-
Environmental sequence PS13G4	GU072526.1	-	-
ex marine sediment metagenome	**SRR8157148**		

Stenophoroidae			
Stenophora nickeli	OP390086.1		
Gregarinoidae			
Didymophyes hydrobiina	PX830639_1	-	-
Gregarina coronata	FJ459743.1	-	PP163374.1
Gregarina niphandrodes	AF129882.1		
Gregarina sp. ex *Phaedon brassicae* (beetle)	JF412715.1		
Hirmocystis ventricosa	PX830636_1	-	-
Leidyana gryllorum	**JBLWGC010000073.1**		
ex *Echmepteryx hageni* (scaly-winged barklice)	**GCWY01008460.1**<br>**GCWY01007447.1**	**SRR1821982**	
Dactylophoroidea			
Echinomera hispida	PP657469.1		
Trichorhynchus efeykini	PP657470.1		
Stylocephaloidae			
Gen. sp. ex *Blaps parvicollis* (beetle)	OQ940287.1		
basal Actinocephaloidea			
Geneiorhynchus manifestus	FJ459739.1	-	-
crown Actinocephaloidea			
Apicystis sp	MF998086.1	-	-
Aranciocystis muskarensis	KU926299.1	-	-
Ascogregarina taiwanensis	EF666482.1		
Farinocystis sp ex *Euscepes postfasciatus* (beetle)	AB748927.1		
Mattesia geminata	AY334568.1	-	-
Ophryocystis elektroscirrha	**JAQIFP010000141.1**		
Paraschneideria metamorphosa	FJ459755.1	-	-
Psychodiella chagasi	FJ865354.1	-	-
ex *Acarus siro* (barn mite)	AY490099.1		
ex *Aedes albopictus* (mosquito)	**SWKY01001477**		
ex *Bombus rufofasciatus* (bumblebee)	**JACAFK010000180.1**		
ex *Caenocrypticoides* sp. (beetle)	**GKFD01064284.1**		
ex *Dendroctonus ponderosae* (beetle)	**APGL01005993.1**		
ex *Galerucella nymphaeae* (beetle)	**CAXBTC010000007.1**		
ex *Larinus minutus* (beetle)	**JBGEDK020000257.1**		
ex *Leptinotarsa lineolata* (beetle)	**WOGR01015389.1**		
ex *Megachile rotundata* (bee)	**JAJHPZ010001165.1**		
ex *Micropterna sequax* (caddisfly)	**JAGUCF010102708.1**		
ex *Prionychus ater* (beetle)	**CBCOQZ010000048.1**		
ex Scatopsidae Gen. sp. GD3 (dung midge)	**GGAV01017349.1**	-	**SRR6155939**
ex *Tinea pellionella* (casemaking clothes moth)	**CANUGQ010000006.1**		
ex *Torymus sinensis* (chalcid wasp)	**JAPYZP010011370.1**		
Ancyrophora noteri	PX830638.1	-	-
Ancyrophora uncinata	PX830637.1	-	-
Apolocystis bosanqueti	OP626897.1	-	-

Coronoepimeritus otoensis	OP354225.1		
Gen. sp. M618 ST7 5 ex *Margaritifera margaritifera*	OR133333.1	-	-
Gen. sp. M818 ST9 6 ex *Margaritifera margaritifera*	OR133340.1	-	-
Hoplorhynchus acanthatholius	FJ459750.1	-	-
Metamera schubergi	PX830632.1	-	-
Monocystis agilis	AF457127.1	**SRR8980208**	
Odonaticola pyriforma	PV932821.1	-	-
Prismatospora evansi	FJ459756.1	-	-
Pyxinia crystalligera	FJ459759.1	-	-
Quadruspinospora mexicana	MK181532.1	-	
Steinina ctenocephali	GU320208.1	-	-
Syncystis mirabilis	DQ176427.1	-	-
ex *Anaplectoides prasina* (green arches)	**CBCRJD010001767.1**		
ex *Carebara altinodus* (ant)	**JBJGMS010000087.1**		
ex *Condylostylus longicornis* (fly)	**JAQSLO010000857.1**		
ex *Junonia stemosa* (butterfly)	**DVST01000001.1**		
ex *Microceris variicolor* (butterfly)	**DWFJ01000003.1**		
ex mine metagenome	**JBAIHO010008244.1**		
ex *Panulirus japonicus* (lobster larva)	AB248106.1	-	-
ex *Podolestes orientalis* (damselfly)	**GCLF01084154.1**	**GCLF01084311.1**	
ex *Popillia japonica* (beetle)	**SMLS01015751.1**		
ex soil metagenome	**JANRHI010058780.1**		
ex wetland metagenome	**JAOQIB010015688.1**		
**Cryptonaiadidae fam. nov. (actinocephalids restricted to freshwater hosts)**			
Gen. sp. M3-18_TEdslCl10:6 ex *Margaritifera margaritifera* (freshwater pearl mussel)	OR167018.1	-	-
Gen. sp. M4-18_TEdslCl11:20 ex *Margaritifera margaritifera* (freshwater pearl mussel)	OR167021.1	-	-
Gen. sp. M1-19_TEdslCl13:17 ex *Margaritifera margaritifera* (freshwater pearl mussel)	OR167184.1	-	-
Cryptonaiada temporaria isolate Rt_E2_D3 (tadpole)	KT717638.1		-
Cryptonaiada temporaria isolate Rd_E7_22L7 (tadpole)	KT717658		-
Cryptonaiada temporaria isolate Nt12+54 (tadpole)	**OZ557594.1**	**-**	**OZ557595.1**
ex *Amphizoa lecontei* (water beetle)	**SRR5892093**		
ex *Enochrus cinctus* (water beetle)	**JAUIMW010012868.1**		
ex *Hydrochara caraboides* (water beetle)	**GDPR01019603.1**	-	**SRR2083674**
