# Supplementary material for: Cellular organization and phylogeny of Cryptonaiada gen. n. (Cryptonaiadidae fam. n.) shed light on the evolution of intracellular vertebrate parasitism in Apicomplexa
Source: mBio. 2026 Oct 5;17(11):e01957-26. doi: 10.1128/mbio.01957-26 (PMC13753809; doi:10.1128/mbio.01957-26)
Supplement: Supplementary file 5 [file mbio.01957-26-s0007.pdf]

Table S2. List of data sources used in phylogenomic analysis in Figure 7.

Organism	Data type	Source
Aggregata octopiana	WGS data	(Mathur et al. 2020) https://doi.org/10.1093/gbe/evaa244
Alexandrium tamarense	Transcriptome	MMETSP0382 (https://zenodo.org/record/3247846)
Ancora sagittata	Transcriptome	(Janouškovec et al. 2019) https://doi.org/10.7554/eLife.49662.001
Ascogregarina taiwanensis	EukProt Transeq translations of WGS data	(Templeton et al. 2010) https://doi.org/10.1093/molbev/msp226 & EukProt (https://doi.org/10.24072/pcjournal.173)
Bigelowiella natans	Genome	https://phycocosm.jgi.doe.gov/Bigna1/Bigna1.home.html
Cafeteria roenbergensis	NCBI genome	GCA_008330645.1
Cardiosporidium cionae	NCBI genome	GCA_015476325.1
Cephaloidophora cf. *communis*	Transcriptome	(Janouškovec et al. 2019) https://doi.org/10.7554/eLife.49662.001
Chromera velia	Genome	https://cryptodb.org/common/downloads/
Colpodella angusta	Transcriptome	(Janouškovec et al. 2019) https://doi.org/10.7554/eLife.49662.001
Colponema vietnamica	Transcriptome	(Tikhonenkov et al. 2020) https://doi.org/10.1016/j.ympev.2020.106839
Colponemidia sp.	Transcriptome	(Tikhonenkov et al. 2020) https://doi.org/10.1016/j.ympev.2020.106839
Cryptonaiada temporaria	Transcriptome	ENA: PRJEB125518
Cryptosporidium muris	NCBI genome	GCF_000006515.1
Cryptosporidium parvum	NCBI genome	GCF_000165345.1
Digyalum oweni	Transcriptome	(Janouškovec et al. 2019) https://doi.org/10.7554/eLife.49662.001
Ectocarpus siliculosus	NCBI genome	GCA_000310025.1
Eimeria falciformis	Genome	https://toxodb.org/common/downloads/
Filipodium phascolosomae	Transcriptome	(Mathur et al. 2021) https://doi.org/10.1016/j.cub.2021.04.028
Gregarina niphandrodes	NCBI genome	GCF_000223845.1
gregarine ex *Helicoverpa* spp.	Transcriptome	NCBI TSA: GBTA00000000, GBDM00000000
gregarine ex *Parafontaria laminata*	Transcriptome	(Yazaki et al. 2021) https://doi.org/10.1016/j.parint.2021.102364
gregarine ex *Scolopocryptops rubiginosus*	Transcriptome	(Yazaki et al. 2021) https://doi.org/10.1016/j.parint.2021.102364
gregarine ex *Symphylella* sp.	Transcriptome	NCBI SRA: SRR1653241
gregarine ex *Teleopsis dalmanni*	Transcriptome	NCBI TSA: GBBP01000000
Heliospora caprellae	Transcriptome	(Mathur et al. 2019) https://doi.org/10.1016/j.cub.2019.07.019
Hematodinium sp.	Transcriptome	NCBI TSA: GEMP01000000

Lankesteria metandrocarpae	Transcriptome	(Mathur et al. 2021) https://doi.org/10.1016/j.cub.2021.04.028
Lecudina tuzetae	Transcriptome	(Mathur et al. 2019) https://doi.org/10.1016/j.cub.2019.07.019
Legerella nova	Transcriptome	(Na et al. 2024) https://doi.org/10.1016/j.ympev.2024.108060
Leidyana gryllorum	NCBI genome	GCA_048772705.1
Lunidium terebellae	Transcriptome	(Mathur et al. 2023) https://doi.org/10.1093/molbev/msad002
Metzidium orientale	Transcriptome	(Mathur et al. 2023) https://doi.org/10.1093/molbev/msad002
Monocystis agilis	Transcriptome	(Mathur et al. 2019) https://doi.org/10.1016/j.cub.2019.07.019
Ophryocystis elektroscirrha	Genome	(Mongue et al. 2023) https://doi.org/10.1186/s12864-023-09350-0
Oxyrrhis marina	Transcriptome	MMETSP0469 (https://zenodo.org/record/257026)
Oxytricha trifallax	NCBI genome	NCBI WGS: AMCR00000000
Perkinsus marinus	NCBI genome	GCF_000006405.1
Piridium sociabile	Transcriptome	(Mathur et al. 2019) https://doi.org/10.1016/j.cub.2019.07.019
Plasmodiophora brassicae	NCBI genome	NCBI WGS: CDSF00000000
Plasmodium falciparum	NCBI genome	GCF_000002765.6
Platyproteum vivax	Transcriptome	(Mathur et al. 2021) https://doi.org/10.1016/j.cub.2021.04.028
Polyplicarium lacrimae	Transcriptome	(Mathur et al. 2023) https://doi.org/10.1093/molbev/msad002
Polyrhabdina sp.	Transcriptome	(Janouškovec et al. 2019) https://doi.org/10.7554/eLife.49662.001
Porospora cf. *gigantea*	Genome	(Boisard et al. 2022) https://doi.org/10.1186/s12864-022-08700-8
Protocruzia adherens	Transcriptome	(Keeling et al. 2014) https://doi.org/10.1371/journal.pbio.1001889 - MMETSP0216
Pterospora schizosoma	Transcriptome	(Mathur et al. 2019) https://doi.org/10.1016/j.cub.2019.07.019
Rhytidocystis sp. ex *Travisia forbesii*	Transcriptome	(Janouškovec et al. 2019) https://doi.org/10.7554/eLife.49662.001
Saprolegnia parasitica	NCBI genome	GCA_000151545.2
Selenidium pygospionis	Transcriptome	(Janouškovec et al. 2019) https://doi.org/10.7554/eLife.49662.001
Siedleckia nematoides	Transcriptome	(Janouškovec et al. 2019) https://doi.org/10.7554/eLife.49662.001
Stentor coeruleus	NCBI genome	GCA_001970955.1
Tetrahymena thermophila	NCBI genome	GCA_000189635.1
Theileria equi	NCBI genome	GCF_000342415.1
Toxoplasma gondii	NCBI genome	GCF_000006565.2
Vitrella brassicaformis	NCBI genome	GCA_001179505.1
